# Development of Multidrug Resistance in Acute Myeloid Leukemia Is Associated with Alterations of the LPHN1/GAL-9/TIM-3 Signaling Pathway

**DOI:** 10.3390/cancers13143629

**Published:** 2021-07-20

**Authors:** Zuzana Kocibalova, Martina Guzyova, Ivana Borovska, Lucia Messingerova, Lucia Copakova, Zdena Sulova, Albert Breier

**Affiliations:** 1 Faculty of Chemical and Food Technology, Institute of Biochemistry and Microbiology, Slovak University of Technology in Bratislava, Radlinského 9, 812 37 Bratislava, Slovakia; zuzana.kocibalova@stuba.sk (Z.K.); martina.guzyova@stuba.sk (M.G.); lucia.messingerova@stuba.sk (L.M.); 2 Department of Biochemistry and Cytochemistry, Institute of Molecular Physiology and Genetics, Centre of Bioscences SAS, Slovak Academy of Sciences, Dúbravská cesta 9, 840 05 Bratislava, Slovakia; ivana.sevcikova@savba.sk; 3 National Cancer Institute, Klenová 1, 833 10 Bratislava, Slovakia; copakova@genvias.sk

**Keywords:** latrophilin-1, TIM-3, galectin-9, ABCB1, CD44, acute myeloid leukemia, multidrug resistance, alternative splicing

## Abstract

**Simple Summary:**

Latrophilin-1 is a latrotoxin receptor and is commonly found in the plasma membrane of neurons. This receptor has recently been identified in the plasma membrane of myeloid leukemia blasts but not in healthy leukocytes. We have shown that the development of drug resistance associated with ABCB1 overexpression leads to decreased regulation of latrophilin-1 in human acute myeloid leukemia cell lines. Here, we proved that latrophilin-1 expression also occurs in the myeloid blasts of patients newly diagnosed with myelodysplastic syndrome when ABCB1 is overexpressed. Furthermore, we provided the evidence that in case of ABCB1 overexpression in human acute myeloid leukemia cell lines, changes in the expression of latrophilin-1-regulated proteins occur that are thought to allow myeloid blasts to escape control of the immune system. All of the above changes in protein expression appear to be involved in the overall altered phenotype of neoplastic myeloid cells following ABCB1-mediated MDR development.

**Abstract:**

P-glycoprotein (known as ABCB1 transporter) expression in myeloid blasts of acute myeloid leukemia (AML) or myelodysplastic syndrome (MDS) leads to the commonly observed multidrug resistance. Overexpression of latrophilin-1 was detected in leukemic cells from AML patients. In a previous study, we showed that ABCB1 overexpression is associated with decreased latrophilin-1 expression in MOLM-13/VCR and SKM-1/VCR AML cell variants derived from MOLM-13 and SKM-1 cells by vincristine selection/adaptation. In the present study, we found that if ABCB1 overexpression occurs in myeloid blasts of newly diagnosed MDS patients, latrophilin-1 expression is attenuated. Latrophilin-1 may initiate TIM-3- and galectin-9-mediated immune escape. We demonstrated changes in the expression of both proteins by comparing ABCB1-positive cell variants (MOLM-13/VCR, SKM-1/VCR) with their ABCB1-negative counterparts. Galectin-9 was present in our cell lines in eight protein isoforms for which we identified the respective transcription variants resulting from alternative splicing, and we verified their structure by sequencing. The isoform profile of galectin-9 was different between ABCB1-positive and ABCB1-negative cell variants. The interaction partner of galectin-9 is CD44, and its expression was altered in the ABCB1-positive variants MOLM-13/VCR and SKM-1/VCR compared to their ABCB1-negative counterparts.

## 1. Introduction

Acute myeloid leukemia (AML) is a heterogeneous severe oncological disorder in which a characteristic clonal expansion and accumulation of abnormally differentiated myeloid blasts in the bone marrow, peripheral blood and other tissues is present. This is caused by a faulty mechanism of hematopoiesis in leukemic-transformed cells, primarily leading to cytopenias with consequent susceptibility to infectious diseases and persistent hemorrhage [1,2,3]. It can develop de novo or progress from other hematological disorders, such as myelodysplastic syndrome (MDS), even after previous cytotoxic chemotherapy [3,4]. Although significant progress has been made in understanding the biology, etiology and pathogenesis of AML, leading to new diagnostic approaches, the standard treatment is still being applied in combination with new therapeutic approaches [5].

The gold standard treatment consists of induction chemotherapy (cytarabine in combination with anthracyclines) to achieve remission, followed by consolidation chemotherapy and, if possible, either allogeneic or autologous stem cell transplantation [2,5,6]. Despite improved genetic and molecular profiling and risk stratification, AML remains a serious disease with an unsatisfactory prognosis, and there is a high relapse rate in patients after the first remission. This could be explained by the leukemia stem cell (LSC) theory in which rare leukemic blasts are thought to be responsible for relapse and for maintaining AML. LSCs are capable of self-renewal and are primarily drug resistant [7].

Intensive experimental and translational research has been conducted over the last 20 years on the use of immunomodulatory agents (e.g., lenalidomide [8]), demethylating agents (e.g., azacytidine and deoxyazacytidine [9,10]), proteasome inhibitors (e.g., bortezomib [11,12]) and histone deacetylase inhibitors (e.g., vorinostat [13]). Therapy with these and other promising drugs is applied in combination with standard intensive therapy, especially in patients with bone marrow malignancies, if the standard treatment alone fails. The need for a combination of multiple treatment approaches stems from the fact that although monotherapy with new targeting agents in selected subgroups of diseases is satisfactory and often more effective and less toxic than conventional treatment, the chances of being curative are small [14]. Therefore, it is crucial to clarify how best to incorporate new drugs into overall treatment strategies, including bone marrow cell transplantation.

Drug resistance is a real obstacle in chemotherapy for cancer patients, including AML. It can occur either intrinsically or can be acquired during/after drug treatment. Its multiple form, i.e., multidrug resistance (MDR), when leukemic blasts are cross-resistant to several structurally and pharmacologically unrelated anticancer agents, is a feature that significantly worsens the patient’s prognosis [15,16]. Particular attention should be given to the increased drug efflux, as the overexpression of drug efflux pumps from the ABC transporter superfamily, especially its ABCB1 member (also known as P-glycoprotein, P-gp), is the most common molecular feature of MDR development [17].

We have shown in previous studies that the development of MDR in leukemic cells mediated by ABCB1 overexpression is associated with multiple and complex changes in the transcriptome, proteome, and cell surface glycome [10,18,19,20,21,22,23].

Recently, expression of the G-protein coupled plasma membrane receptor latrophilin-1 (LPHN1, an *ADGRL1* gene product in humans) has been demonstrated in leukemic blasts and primary human hematopoietic stem cells positive for CD34 but lacking in healthy mature leukocytes [24,25,26]. The authors of these studies hypothesized that the expression of latrophilin-1 in neoplastic leukemia cells could be important as a diagnostic tool in predicting patient prognosis.

LPHN1 is the mammalian plasma membrane receptor known to bind α-latrotoxin (a venom component of *Latrodectus mactans*, also known as the black widow spider) as a xenoligand. This receptor is typically expressed in neuronal cells (but not in glia), where it co-modulates calcium-dependent neurotransmitter release [27,28]. Apart from the abovementioned expression of LPHN1 in leukemic myeloid blasts, there are few and often contradictory findings about the expression of this receptor in neoplastic cells [29]. We previously demonstrated LPHN1 expression in AML SKM-1 and MOLM-13 cells [30]. However, we showed that LPHN1 expression is downregulated (compared to parental cells) in cell variants of both AML lines (SKM-1/VCR and MOLM-13/VCR) that expressed ABCB1. These sublines were established through long-term cultivation with increasing concentrations of vincristine (VCR). Therefore, we conclude that the development of MDR in these cells induces changes in LPHN1 expression. Nevertheless, LPHN1 expression, when estimated in patient samples, may provide important additional information about the status of AML cells.

LPHN1 function in AML cells remains unclear. It was proposed as one of the regulatory molecules of the immune escape mechanism. Activation of LPHN1 leads to the upregulation of both galectin-9 (GAL-9) and TIM-3 (encoded by *HAVCR2* gene) [24], which together form an autocrine loop that can drive the self-renewal capacity of leukemic blasts [31]. GAL-9 is a member of the mammalian lectin family and was originally discovered as an eosinophil chemoattractant and is currently mostly described as a potent immunomodulatory molecule that can either induce apoptosis in cytotoxic T cells or disrupt the production of cytokines in NK cells [32]. Expression of the *LGALS9* gene (encoding GAL-9) produces several isoforms that are synthesized using various transcript splice variants [33,34,35]. However, information on the existence of these isoforms in AML cells is lacking. TIM-3 is a receptor for GAL-9 and is its trafficking protein, since GAL-9, similar to other galectins, lacks the signal peptide for extracellular release [24,32]. In addition, TIM-3 is known to be one of the coinhibitory receptors in immune system escape mechanisms [36].

In this study, we focused on the following objectives: (i) To verify the relationship between LPHN1 and ABCB1 expression in clinical samples from MDS patients and determine whether there is a relationship between the expression levels of LPHN1 and ABCC1, which is another member of the MDR-causing ABC transporter family; (ii) to evaluate the expression profile of GAL-9 and TIM-3 in the leukemic cell lines SKM-1 and MOLM-13 and their ABCB1-positive variants SKM-1/VCR and MOLM-13/VCR; and (iii) to identify splice variants of *LGALS9* gene transcripts and to assign appropriate isoforms of the GAL-9 protein.

## 2. Materials and Methods

### 2.1. Clinical Samples of Patients with MDS

In this retrospective study, the expression of *ADGRL1, ABCB1* and *ABCC1* was examined in mononuclear blast cells of patients with MDS. Peripheral blood was obtained from 17 patients (7 women, 10 men; age 48–84 years; median age 65 ± 9.7 years) with MDS at the National Cancer Institute, Bratislava, Slovakia between June 2011 and October 2013 after approval by the Institutional Ethics Board. Peripheral blood was collected at the time of diagnosis without any previous treatment. Peripheral blood mononuclear blast cells (PBMCs) were isolated using Ficol Paque Plus (ProScience Tech. s.r.o., Slovakia) via gradient centrifugation of the patient’s peripheral blood [37]. 

### 2.2. Cell Lines and Cultivation Conditions

As an experimental model, the following two human acute myeloid leukemia cells were used in this study: SKM-1 (ACC 547)—derived from the peripheral blood of a 76-year-old Japanese male with acute myeloid leukemia (AML M5 according to the FAB classification) that developed after previous MDS; and MOLM-13 (ACC 554)—derived from the peripheral blood of a 20-year-old man with acute myeloid leukemia (AML M5a according to the FAB classification) at relapse; in this patient, AML developed after previous MDS. Both cell lines were supplied by Deutsche Sammlung von Mikroorganismen und Zellkulturen GmbH, Germany. Detailed characteristics of MOLM-13 and SKM-1 cells, including karyotype analysis are given on the supplier’s website or are available in [38]. The drug-resistant cell lines SKM-1/VCR and MOLM-13/VCR overexpressing ABCB1 drug transporter were established by long-term cultivation with the anticancer agent vincristine (VCR; Sigma Aldrich, St. Louis, MO, USA). The VCR concentrations were increased stepwise to a final concentration of 60 nM, as previously described [18,20]. All cell lines were cultivated in RPMI-1640 medium supplemented with 12% fetal bovine serum (both purchased from Gibco, Miami, OK, USA), 100,000 units/mL penicillin and 50 mg/L streptomycin (both purchased from Sigma Aldrich) for 48 h at 37 °C in a humidified atmosphere containing 5% CO_2_.

### 2.3. Determination of ADGRL1, LGALS9, HAVCR2, CD44, ABCB1, ABCC1 and ACTB Transcript Expression in Cell Lines and Patient Samples by RT-PCR and qRT-PCR

The expression of all genes of interest was measured by RT-PCR with subsequent agarose gel electrophoresis and/or by qRT-PCR. Total RNA from patient samples and cell lines was isolated by Tri Reagent (MRC, Cincinnati, OH, USA) according to the manufacturer’s protocol. cDNA synthesis was performed by using a RevertAid^TM^ H Minus First-Strand cDNA synthesis kit (Thermo Fisher Scientific, Waltham, MA, USA) according to the manufacturer’s protocol with DNase-treated RNA as template. PCR was performed using a DreamTaq PCR kit (Thermo Fisher Scientific) according to the manufacturer’s instructions in a total volume of 25 µL. PCR thermal cycling conditions were as follows: initial denaturation (95 °C, 3 min; 1×); denaturation (95 °C, 30 s), annealing (T_A_; 30 s), extension (72 °C, 1 min; number of cycles 30); and final extension (72 °C, 5 min). The primer sequences and annealing temperatures (T_A_) used in this study are summarized in Supplementary Table S1. PCR products were separated on 1.5% TAE-agarose gels (Lonza, Rockland, ME, USA) containing GelRed^TM^ nucleic acid gel stain (Biotium, Fremont, CA, USA) in TAE electrophoresis buffer and visualized by an Amersham Imager 600 (GE Healthcare, Chicago, IL, USA). Densitometric analysis of the respective bands was performed by ImageJ (version 151j8, National Institutes of Health, Bethesda, MD, USA) and normalized to *ACTB* levels. All experiments were repeated at least 3 times [18,20].

qRT-PCR was used to quantify *HAVCR2, LGALS9* and *ABCB1* mRNA levels in both sensitive and resistant cell lines, while *ACTB* was used as an internal loading control. Total RNA isolation and cDNA synthesis were performed as described above. PCR was performed using TaqMan^TM^ Universal Master Mix II, no UNG (Thermo Fisher Scientific) according to the manufacturer’s protocol. The following TaqMan assays were used in this study: *HAVCR2*—HS00958618_m1; *LGALS9*—Hs04190742_mH; *ABCB1*—Hs00184500_m1; *ACTB*—Hs01060665_g1. The thermal cycling conditions were as follows: polymerase activation (95 °C, 10 min); PCR—40 cycles: denaturation (95 °C, 15 s) and annealing/extension (60 °C; 1 min). The relative expression of individual genes was evaluated by the Livak method (2^−ΔΔCT^) [10].

### 2.4. Alternative Splicing of LGALS9 and Dataset of Splice Variants

To detect *LGALS9* transcript variants because of alternative splicing, a special pair of primers was used in this study (for the detection of TV1, X1, TV2, X2, X3, TV4 and X4): F: 5′-CCATCTCCGTCAATGGCTCT-3′ and R: 5′-CACCTTGAGGCAGTGAGCTT-3′ with T_A_ = 58 °C. This pair of primers produced 7 PCR products with distinct molecular weights. For detection of the X5 variant, another pair of primers was designed to amplify a single 288 bp PCR product: F: 5′-CACTGCCACAGTGACCTTCT-3′ and R: 5′-GGCAATTAGGCATGTGCTCG-3′ with T_A_ = 58 °C. After RT-PCR, all PCR products were separated by gel electrophoresis on a 2% agarose-TAE gel in TAE buffer. After separation, the PCR products were cut from the gel and purified with a GeneJET Gel Extraction Kit (Thermo Fisher Scientific). The sequence of each PCR product of the respective transcript variants was determined by Sanger sequencing (Eurofins Genomics, Eurofins Scientific, Luxembourg). The obtained sequences were analyzed by BLASTN (National Institutes of Health). Additional alignment analysis was performed using Clustal Omega (European Bioinformatics Institute, Hinxton, UK) [39]. Densitometric analysis of the respective bands was performed with ImageJ and normalized to *ACTB* levels. All experiments were repeated at least 3 times.

All transcript variants and isoforms of galectin-9 structure and sequences were obtained from the NCBI gene database (National Institutes of Health); gene ID *LGALS9*: 3965. The respective IDs (accession numbers) of transcripts and isoforms are listed in Table 1. The intronic sequence (intron 5–6) of the full-length (FL) variant was obtained from the Ensembl Database (European Bioinformatics Institute, Hinxton, UK); ID *LGALS9* ENSG00000168961, transcript ID FL ENST00000395473.7 [40].

### 2.5. Detection of Proteins by Western Blotting

The protein expression of GAL-9, TIM-3, CD44 and ABCB1 was analyzed by Western blotting, and GAPDH was used as an internal loading control, as previously described [18,30]. Cells were lysed in lysis buffer (50 mM Tris-HCl, 5 mM EDTA, 150 mM NaCl, 0.5% Nonidet P-40; pH 8) supplemented with protease inhibitor cocktail (Sigma Aldrich) according to the manufacturer’s protocol. Equal amounts (40 µg per lane) of protein lysates were denatured by heating at 95 °C for 5 min in SDS-PAGE sample buffer, loaded on 12% Tris-glycine gels and separated by SDS-PAGE using a Mini-PROTEAN^®^ Tetra Cell System (Bio-Rad Laboratories, Philadelphia, PA, USA). Subsequently, proteins were transferred to nitrocellulose membranes using a Trans-Blot^®^ SD Semi-Dry Transfer Cell (Bio-Rad Laboratories). The membranes were then incubated in 3% BSA or 3% nonfat milk in PBST or TBST (PBS or TBS, 1% (*v*/*v*) Tween-20) for 1 h at RT. The membranes were incubated with the following primary antibodies at 4 °C overnight: GAPDH (Merck Millipore, Burlington, MA, USA), ABCB1, GAL-9, TIM-3 and CD44 (all from Abcam, Cambridge, UK). After washing 3 times with PBST or TBST, anti-rabbit or anti-mouse antibodies conjugated with horseradish peroxidase (Santa Cruz Biotechnologies, Dallas, TX, USA) was used as a secondary antibody. The protein bands were visualized by SuperSignal^TM^ West Pico PLUS Chemiluminescent Substrate (Thermo Fisher Scientific) using an Amersham Imager 600 (GE Healthcare). Densitometric analysis of the respective bands was performed with ImageJ and normalized to GAPDH levels. All experiments were repeated at least 3 times [18,30].

### 2.6. Statistical Analysis

All experiments were repeated at least 3 times, and data represent the mean value ± standard error of the mean (SEM). Statistical significance was evaluated by unpaired Student’s *t*-test using Excel 2016 (Microsoft Corporation, Redmond, WA, USA), GraphPad Prism 8.2.1 (GraphPad Prism Software for Windows, San Diego, CA, USA) and/or SigmaPlot 8.02 (Systat Software, Inc., San Jose, CA, USA).

## 3. Results

### 3.1. Latrophilin-1 Is Downregulated in Clinical Samples of Myelodysplastic Syndrome (MDS) with ABCB1 and ABCC1 Expression

First, we wanted to verify whether there is a reciprocal relationship between the gene expression of *ABCB1* and *ADGRL1*, which we found in the AML cell lines SKM-1 and MOLM-13 in samples from patients with hematological malignancies. Both cell lines are derived from patients whose disease developed from MDS [41,42], and these lines are widely used as models of MDS [38].

**Figure 1:**
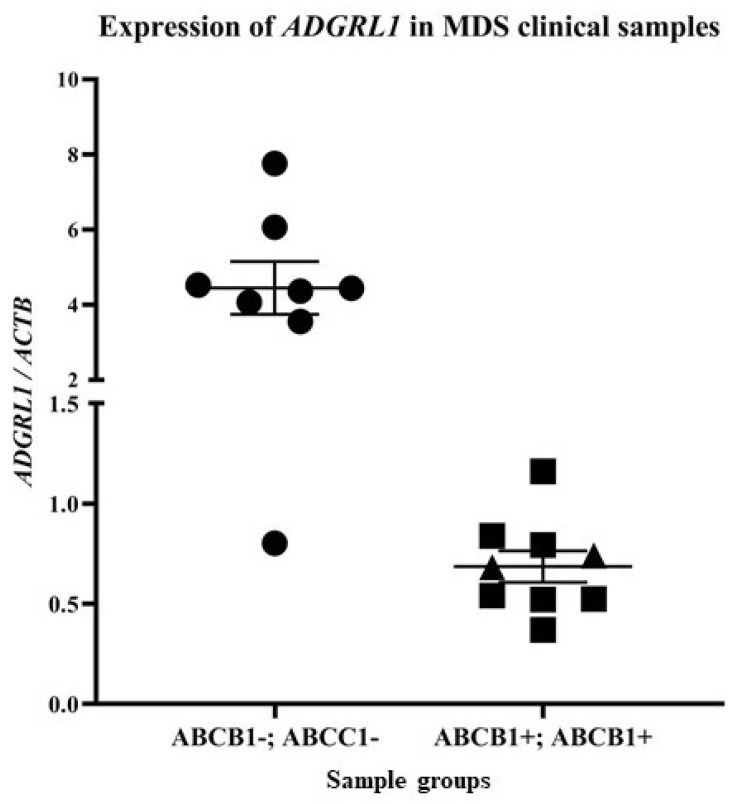
Detection of *ADGRL1* mRNA expression by RT-PCR in samples from patients with MDS with and without expression of the ABC transporters (*ABCB1* and *ABCC1*) involved in multidrug resistance. Data represent the mean ± SEM for each statistical sample group. Data for *ABCB1+/ABCC1+* cases differ significantly from the *ABCB1−/ABCC1−* at the level *p* ≤ 0.001. *ABCB1−/ABCC1−* cases are depicted by circles, *ABCB1+/ABCC1+* cases by squares, while samples with only *ABCC1+* are depicted by triangles.

Therefore, in a group of 17 newly diagnosed patients with MDS without any prior treatment, we focused on detecting possible changes in *ADGRL1* gene expression in relation to *ABCB1* gene expression. We also determined the expression of the *ABCC1* gene, which encodes the multidrug resistance-associated protein MRP1, another plasma membrane drug efflux pump [43]. We detected *ADGRL1* expression in all 17 cases (Figure 1). In seven samples, we found the coexpression of the drug transporter genes (*ABCB1, ABCC1*), and in the other two cases, only *ABCC1* expression was found. In the remaining eight cases, we did not observe the expression of these drug transporters.

Densitometric quantification of the respective PCR product bands allowed us to compare the means and medians of *ADGRL1* expression in samples negative or positive for ABC transporters (Figure 1). The mean value of *ADGRL1* expression was 4.445 ± 0.705 with a median of 4.398 in samples negative for the expression of both transporters, while the mean value was 0.679 ± 0.102 with a median of 0.541 in samples positive for *ABCB1* expression. Such massive downregulation (more than six-fold) was also observed in *ABCC1*-positive cases, with a mean value of 0.686 ± 0.078 and a median of 0.682. It should be emphasized that the expression of *ADGRL1* in samples positive for the expression of drug transporters did not exceed 1.5 in any case. In contrast, among the drug-negative samples, only one had a low level of *ADGRL1* expression (less than 1.5), and in the other seven samples, the expression of this gene was higher than three.

These data demonstrated that *ADGRL1* expression is also downregulated in clinical samples of myeloid blasts from MDS patients when *ABCB1* and/or *ABCC1* expression is upregulated.

### 3.2. Expression of GAL-9, TIM-3, CD44, ABCB1 and ABCC1 in SKM-1 and MOLM-13 Cells and Their Resistant Counterparts

Consistent with our previous results [30], massive *ABCB1* gene expression was detected only in the resistant sublines (SKM-1/VCR and MOLM-13/VCR) by either RT-PCR or qRT-PCR (Figure S3 in supplementary files). ABCB1 protein was also identified by Western blot exclusively in SKM-1/VCR and MOLM-13/VCR cell variants and not in the parental SKM-1 and MOLM-13 cells (Figure S3 in the supplementary files).

There is strong evidence that LPHN1 in leukemic cells regulates the expression and subsequent release of GAL-9 and TIM-3 into the extracellular matrix [24]. Since our previous results demonstrated a reduction in LPHN1 levels in AML cells expressing ABCB1, we further focused on the detection of GAL-9 (*LGALS9*) and TIM-3 (*HAVCR2*) expression in SKM-1 and MOLM-13 cells and in their ABCB1-positive variants resistant to VCR, (SKM-1/VCR and MOLM-13/VCR). In addition, we examined the expression of a potential interacting receptor of GAL-9—CD44 [35,44,45,46], which is also known as phagocytic glycoprotein-1 [47].

**Figure 2:**
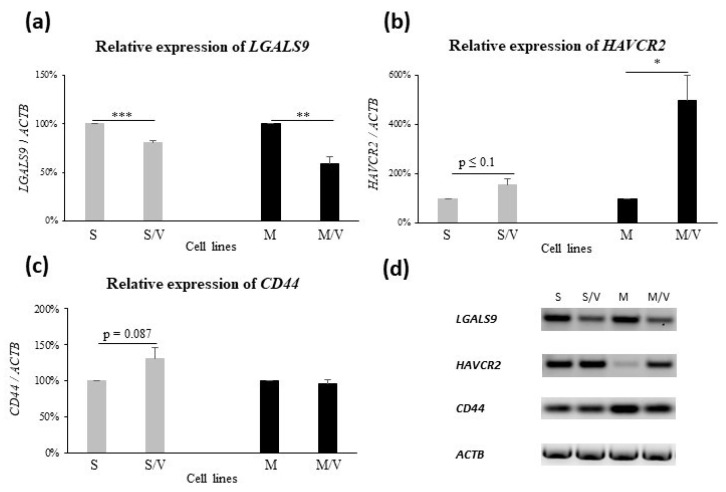
Expression of *LGALS9* (**a**) and *HAVCR2* (**b**) by qRT-PCR and *CD44* detection by RT-PCR (**c**); (**d**)—representative agarose gel of PCR products for all analyzed genes. Data from qRT-PCR represent the mean ± SEM of 4 independent experiments in which all samples were measured at least in duplicate. Data from RT-PCR represent the mean ± SEM of 4 independent experiments. Observed alterations in expression in resistant cell lines in comparison with their sensitive counterparts were statistically significant: * *p* ≤ 0.05; ** *p* ≤ 0.01; *** *p* ≤ 0.001. S—SKM-1; S/V—SKM-1/VCR; M—MOLM-13; M/V—MOLM-13/VCR.

We observed downregulation of the *LGALS9* transcript in both SKM-1/VCR- and MOLM-13/VCR-resistant sublines compared to the corresponding sensitive parental cells (Figure 2a). In contrast, expression of the *HAVCR2* gene, encoding TIM-3, showed the opposite trend. Cell variants overexpressing the *ABCB1* gene (SKM-1/VCR, MOLM-13/VCR) also expressed *HAVCR2* to a greater extent (Figure 2b). While the increased expression of this gene in SKM-1/VCR cells compared to SKM-1 cells was only marginally significant (*p* ≤ 0.1), massive upregulation of *HAVCR2* was seen in MOLM-13/VCR cells (*p* ≤ 0.05) compared to parental MOLM-13 cells. However, we did not observe any changes in *CD44* mRNA levels between MOLM-13 and MOLM-13/VCR cells. However, this gene was slightly upregulated in SKM-1/VCR cells compared to the original SKM-1 cells, but the level of probability (*p* = 0.087) indicated only marginal significance (Figure 2c).

**Figure 3:**
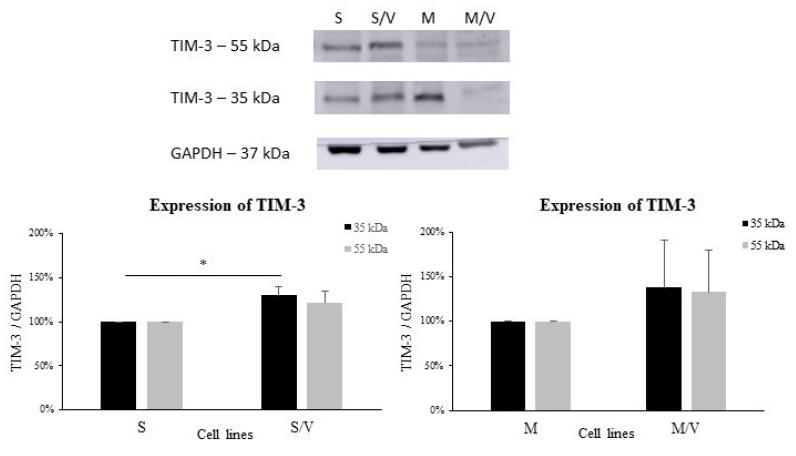
Protein expression of TIM-3. Upper panel—representative Western blot. Lower panel—densitometric analysis of protein bands (35 and 55 kDa) in SKM-1 and SKM-1/VCR (**left**) and MOLM-13 and MOLM-13/VCR (**right**). Upregulation in the resistant cell line was statistically significant (* *p* ≤ 0.05) only for the 35 kDa band in SKM-1/VCR cells in comparison to the parental cell line. Data represent the mean ± SEM of 3 independent experiments. S—SKM-1; S/V—SKM-1/VCR; M—MOLM-13; M/V—MOLM-13/VCR.

**Figure 4:**
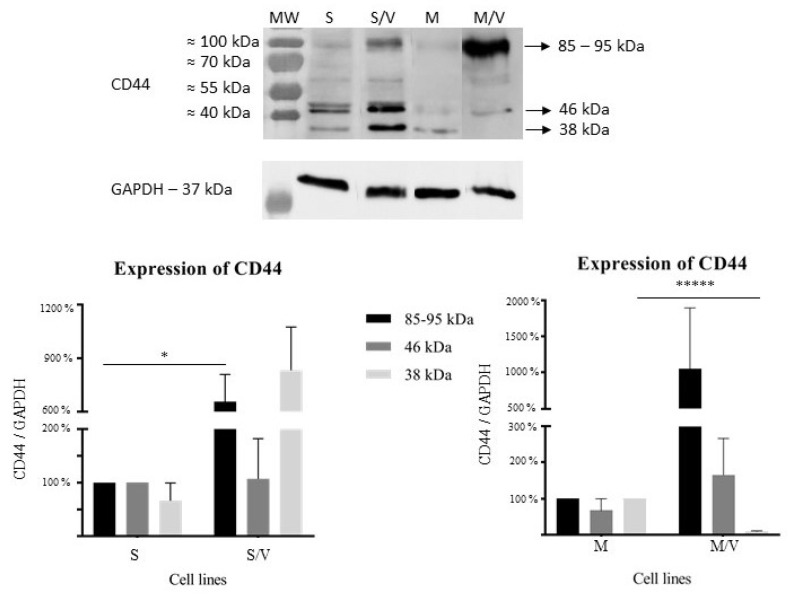
Protein expression of CD44. Upper panel—representative Western blot. Lower panel—densitometric analysis of all 3 protein bands (38, 46 and ≈ 85–95 kDa) in SKM-1 and SKM-1/VCR (**left**) and MOLM-13 and MOLM-13/VCR (**right**). Data represent the mean ± SEM of 3 independent experiments. Statistical significance as follows: * *p* ≤ 0.05; ***** *p* ≤ 0.000001. MW—marker of molecular weight; S—SKM-1; S/V—SKM-1/VCR; M—MOLM-13; M/V—MOLM-13/VCR.

Furthermore, we studied whether either TIM-3 (Figure 3) or CD44 (Figure 4) is upregulated at the protein level in both ABCB1-positive drug-resistant cell variants compared to their sensitive counterparts. These experiments yielded results consistent with the gene expression results obtained from RT-PCR (Figure 2).

In the case of TIM-3, we found two bands with distinct molecular masses of 35 kDa and 55 kDa. The molecular forms may correspond to TIM-3 isoforms, as will be explained in the discussion section. However, both protein bands were upregulated in resistant cell lines (Figure 3) compared to the corresponding parental lines SKM-1 and MOLM-13. The increased expression of the 35 kDa fragment was statistically significant in SKM-1/VCR (vs. SKM-1, *p* ≤ 0.05).

We further identified three different bands of the CD44 protein with molecular weights of 38 kDa, 46 kDa and 85–95 kDa (Figure 4). We hypothesized that these bands are isoforms of CD44, the existence of which will be explained in the Discussion section. In SKM-1/VCR cells, we observed the massive upregulation of 38 kDa band, while in parental cells, this band was seen only two times out of three independent experiments. A similar upregulation was also observed for the 85–95 kDa band in the resistant cell line SKM-1/VCR when compared to the parental line (*p* ≤ 0.05), while the 46 kDa band remained unchanged.

The MOLM-13 cell line had a distinct expression profile. Consistent with SKM-1/VCR, massive upregulation of the 85–95 kDa band was observed in MOLM-13/VCR compared to MOLM-13. The observed expression changes were not statistically significant; however, the upregulation increased with time of cultivation with VCR and the upregulation of *ABCB1* (data not shown). Upregulation in resistant cells also occurred in the case of the 46 kDa protein band, while in the parental cell line, it was observed in two out of three experiments. In contrast, massive and statistically significant downregulation (*p* ≤ 0.000001) was observed for the 38 kDa protein band.

### 3.3. Alternative Splicing of LGALS9 Pre-mRNA in SKM-1 and MOLM-13 Cells

Several lines of evidence suggest the existence of GAL-9 in multiple protein isoforms that result from alternative splicing of pre-mRNA transcribed from the *LGALS9* gene [33,34,48]. However, to date, there has been no investigation of the alternative splicing of GAL-9 in leukemic blasts. Thus, we addressed this issue in another series of experiments using the SKM-1 and MOLM-13 cell lines and their ABCB1-positive counterparts.

**Figure 5:**
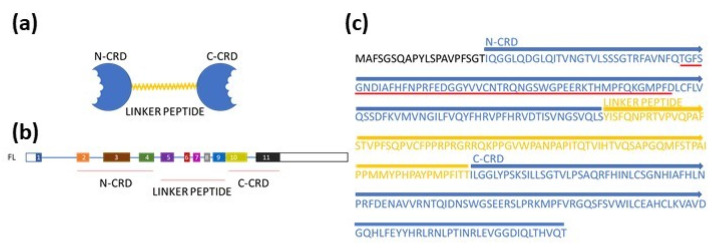
Detailed protein structure of full-length (FL) GAL-9. (**a**) FL protein contains N-terminal CRD (N-CRD) and C-terminal CRD (C-CRD) linked by a linker peptide. N-CRD is encoded by exons 2, 3 and 4 (**b**) and contains 123 AAs (**c**). Linker peptides composed of 87 AAs are encoded by exons 5–9. Exons 10–11 encode the C-CRD, which is composed of 125 AAs. The commercially available anti-GAL-9 antibody used in this study was obtained by immunization with recombinant oligopeptide whose primary structure is underlined in red.

GAL-9 is a tandem repeat protein that contains two carbohydrate recognition domains located at the N- and C-termini (N-CRD and C-CRD), which are linked by a linker peptide (Figure 5).

**Figure 6:**
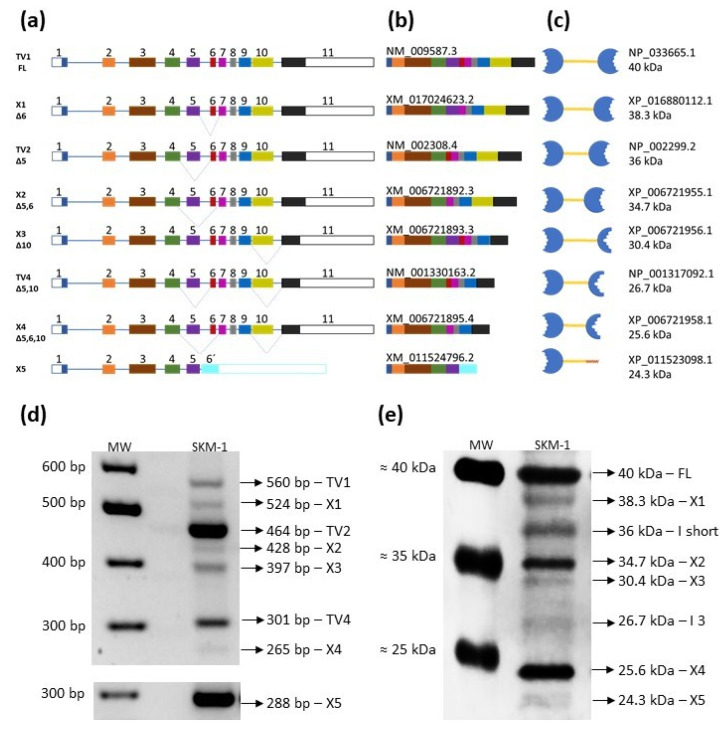
Overview of multiple variants of GAL-9 owing to alternative splicing of LGALS9 pre-mRNA. (**a**) Scheme of alternative splicing of exons 5, 6 and 10 in the various combinations. (**b**) structure of mature mRNA with identification from NCBI gene database; (**c**) structure of respective proteins with identification from the NCBI protein database. Almost every isoform (except variant X5) contains 2 CRDs (blue) that are connected by linker peptides (yellow). Alternative splicing of exons 5 and 6 affects the length of the linker peptide, while splicing of exon 10 leads to truncation of the second (C-terminal) CRD. TV X5 contains alternative exon 6′, which contains a premature stop codon (TAA); thus, X5 comprises only 1 CRD and the peptide tail (protein structure predicted). FL—full-length GAL-9, which is encoded by all 11 exons; (**d**) representative gel of *LGALS9* mRNA PCR products detected by RT-PCR; (**e**) expression of respective GAL-9 protein isoforms detected by Western blot. The expression profiles of transcript variants and isoforms were similar for SKM-1 and MOLM-13 (data not shown). MW—marker of molecular mass; I—isoform.

The human *LGALS9* gene consists of 11 exons (designated 1–11), of which 5, 6 and 10 can alternatively be spliced either alone or in different combinations (Table 1) [34]. As a result of this variability, *LGALS9* pre-mRNA can be further processed into eight different transcript variants (TVs) (according to the NCBI gene database), which can then be translated into eight protein isoforms. Three isoforms are often described in the literature, and only a few papers have recognized six isoforms of GAL-9 (e.g., studies by the Thijssen group [33,34,48]). Splicing of exons 5 and 6 may affect the length of the linker segment, while splicing of exon 10 may lead to premature arrest due to the formation of a new stop codon and shortening of the C-CRD. The seven *LGALS9* TVs are the result of a combination of alternative splicing of these three exons during the conversion of pre-mRNA to mRNA. Furthermore, the final TV (X5) contains the first five exons occurring in all other TVs and new exon 6′, which is typical only for this variant (Figure 6a).

**Figure 7:**
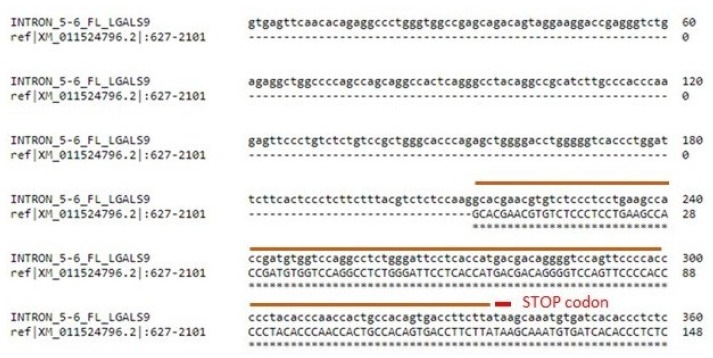
Partial alignment (Clustal Omega) of intron 5–6 from FL LGALS9 (sequence obtained from Ensembl, ID ENSG00000168961, transcript ID of FL ENST 00000395473.7) and exon 6′ from the X5 variant (obtained from the NCBI gene database). Intron 5–6 of FL is 1693 NTs long, while exon 6′ is 1475 NTs. Exon 6′ is almost completely identical intron 5–6, except for the first 212 NTs and the last 6 NTSs, which are probably spliced out in X5. The TAA stop codon (red) probably leads to the truncation of X5. For further details, see additional comparison and alignment analyses in Figures S4–S5 (Supplementary data).

To study the alternative splicing, special primer pairs were designed to detect seven TVs of *LGALS9*, which ensured the generation of PCR products with sizes typical for individual TVs (see Materials and Methods and Figure S1 in the supplementary files). We focused on the X5 TV. As mentioned above, X5 contains the first five exons analogous to all other TVs, but a new exon 6′ appeared, which is typical only for X5. Alignment analysis using Clustal Omega showed that exon 6′ is probably a retained portion of the intron between exons 5 and 6 (intron 5–6). The first 212 nucleotides of intron 5–6 are spliced as an intron sequence, and the remainder consists of the coding sequence of exon 6′, which is only 123 nucleotides. This coding sequence is terminated by a premature stop codon, TAA (Figure 7). Therefore, isoform X5 presumably has a truncated linker peptide and lacks the entire C-CRD. However, further proteomics analysis is needed.

For these reasons, we had to use a special pair of primers for the last eighth TVs in which both forward and reverse primers landed on the predicted exon 6′ (Figure S1 in supplementary files). Further comparison and alignment analyses are shown in Figures S4 and S5. We verified the existence of all eight TVs in SKM-1 cells as a model. Figure 6b schematically depicts the shortening of TVs due to different alternative splicing, and Figure 6d shows a typical agarose gel after electrophoresis of PCR products, where we verified their predicted size. The identity of all TVs was confirmed by sequencing the respective PCR products (Table S3 in the supplementary files). To the best of our knowledge, this was the first identification of all eight TVs of the *LGALS9* gene in acute myeloid leukemia cells (Figure 6d).

The translation process from these eight TVs yields eight protein variants with a specific truncation compared to FL that corresponds to the missing portions in the individual mature mRNA TVs (Figure 6c). The commercial antibody used in this study to detect GAL-9 isoforms in leukemic cells was raised against an antigen containing 50 amino acids (AA 50–100 FL, red underline in Figure 5c), which are present in all eight isoforms. The typical Western blot in Figure 6e verifies the presence and predicted molecular mass of all eight variants of the *LGALS9* gene products.

**Figure 8:**
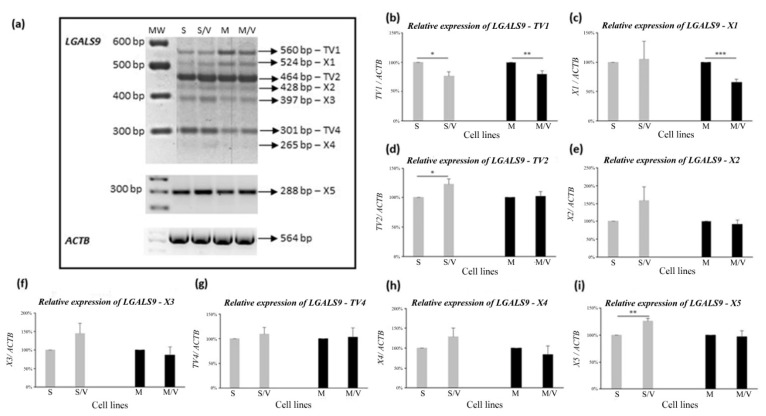
Expression of *LGAL9* transcript variants in SKM-1, SKM-1/VCR, MOLM-13 and MOLM-13/VCR cells. (**a**) Representative gel (from 4 independent experiments) of RT-PCR detection of *LGALS9* TVs in AML cell lines. All TVs (except X5) were amplified by the same pair of primers designed to produce PCR products with distinct molecular weights; (**b**–**i**)—densitometric quantification of respective PCR product bands obtained by RT-PCR of *LGALS9* gene expression in respective cell types. Data represent the mean ± SEM of 4 independent experiments. Statistical significance is as follows: * *p* ≤ 0.05; ** *p* ≤ 0.02; *** *p* ≤ 0.001. MW—molecular weight marker; 1—SKM-1; 2—SKM-1/VCR; 3—MOLM-13; 4—MOLM-13/VCR.

Because we have shown that all *LGALS9* TVs and their related protein variants are present in SKM-1 cells, we focused on studying the detection of these TVs and the corresponding proteins in MOLM-13, SKM-1/VCR and MOLM 13/VCR cells. Here, we report that not only is the global expression of *LGALS9* transcripts altered, but changes in individual TVs and corresponding proteins in the respective cells were also detected. Typical agarose gels showing the PCR products of the eight TVs in SKM-1 and MOLM-13 cells and their ABCB1-positive variants are shown in Figure 8a, and the densitometric quantification of individual TVs in all four cell types is shown in Figure 8b–i.

Densitometric analysis of PCR products of TVs obtained by transcription of the *LGALS9* gene revealed expression changes between sensitive parental cell lines and drug-resistant ABCB1-positive lines. We observed significant downregulation of TV1 (for the full-length protein) in the two resistant lines SKM-1/VCR (*p* ≤ 0.05) and MOLM-13/VCR (*p* ≤ 0.02) compared with sensitive counterpart cells (Figure 8b). In the case of the X1 variant (Figure 8c), significant downregulation (almost 2-fold) was detected in MOLM-13/VCR cells compared to parental cells. However, the cell variants SKM-1 and SKM-1/VCR did not differ significantly in the expression of X1 TV. In contrast, the expression of TV2 was upregulated in SKM-1/VCR (*p* ≤ 0.05 compared to SKM-1), while the levels of this transcript in MOLM-13 and MOLM-13/VCR were almost identical (Figure 8d). Expression of the X5 variant was upregulated in SKM-1/VCR cells (*p* ≤ 0.02 vs. SKM-1), while no changes were found in MOLM-13 cells (Figure 8i). Although there were differences in expression between ABCB1-negative cells and their ABCB1-positive counterparts in the expression of TVs X2, X3, TV4, and X4 (in some cases), these differences did not meet the criteria for statistical significance.

**Figure 9:**
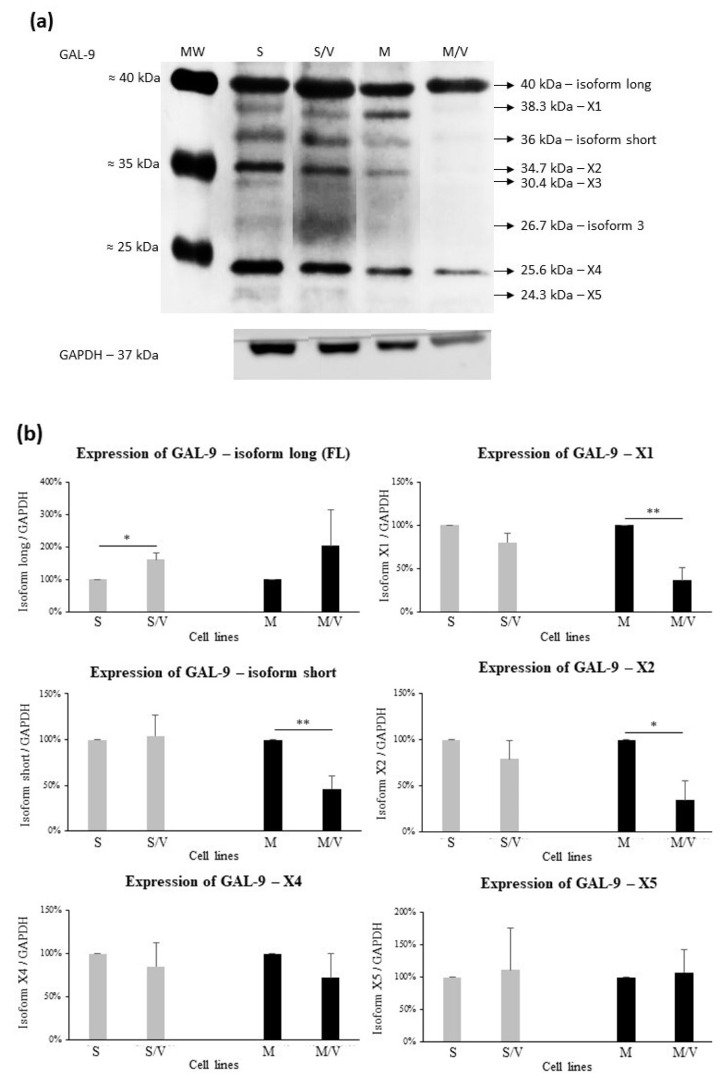
Protein expression of the respective GAL-9 isoforms. (**a**) Representative Western blot; (**b**) graphical comparison of isoform expression levels after densitometric analysis of 6 isoforms (X3 and isoform 3 excluded). Data represent the mean ± SEM of at least 3 independent experiments. Statistical significance is as follows: * *p* ≤ 0.05; ** *p* ≤ 0.02. MW—molecular weight marker; S—SKM-1; S/V—SKM-1/VCR; M—MOLM-13; M/V—MOLM-13/VCR.

Subsequently, we assessed the expression of individual isoforms of GAL-9 protein variants by Western blot in all cell types (Figure 9a). The signals for isoform X3 and isoform 3 were either weak or absent in the different cell types (Figure 6e and Figure 9a) and showed stochastic variability in repeated experiments and were therefore excluded from further quantification. Although isoforms 3 and X3 were present in our leukemic cells, they were present in small amounts and varied among the independent experiments; therefore, they cannot be reliably quantified.

However, the assessment of individual protein isoforms revealed few discrepancies in comparison to mRNA profiling (Figure 9b). Although we observed the downregulation of TV1 mRNA in resistant cells, protein upregulation was found by Western blot for SKM-1/VCR (*p* ≤ 0.05 vs. SKM-1) and for MOLM-13/VCR vs. MOLM-13, but the difference was not significant. In contrast, significant downregulation of X1 TV mRNA in MOLM-13/VCR cells (vs. MOLM-13, Figure 8c) was also confirmed at the protein level (*p* ≤ 0.02, Figure 9b). Altered expression in variants overexpressing the ABCB1 drug transporter was observed for isoform X2 (MOLM-13/VCR vs. MOLM-13, *p* ≤ 0.02) and X4. Isoform short (which is a protein product of TV2) was reduced in MOLM-13/VCR (vs. MOLM-13, *p* ≤ 0.02), although in SKM-1 and its drug-resistant counterpart, no changes were found. Furthermore, the X5 isoform level was only slightly increased in the two ABCB1-positive cell lines, which is consistent with the mRNA expression profile.

## 4. Discussion

Acute myeloid leukemia is a serious oncological disorder with a poor prognosis. Frequent gene mutations and chromosomal aberrations found in myeloid leukemic blasts play a role in the development and acuity of this disease [1]. In recent years, the AML treatment protocol has been optimized, and several promising agents have been introduced to target the regulatory and metabolic pathways involved in the ability of leukemic cells to avoid cell death or escape the immune system.

However, a serious complication of AML chemotherapy is the development of chemoresistance [17], particularly the resistant nature of leukemic stem cells capable of reconstituting the disease during remission and causing relapse of AML [49]. Chemoresistance, especially multidrug resistance, is a phenotype caused by complex molecular functions that affect a wide range of cellular regulatory and metabolic mechanisms. MDR is associated with changes in protein expression that may ensure the survival of cells attacked by cytotoxic drugs, and these changes are often due to an altered trace of DNA methylation. Growing evidence suggests that cells with improper DNA methylation may be suppressed by hypomethylating agents alone or in combination with other drugs (review [50]). This approach is used in acute myeloid leukemia and myelodysplastic syndromes, as well as in other tumors. Although the use of hypomethylating agents is useful, it can lead to resistance against this treatment [10,50]. The ABCB1 drug transporter is considered to be a common cause of chemoresistance in many cancers, including AML [51]. Its overexpression may be accompanied by several changes in the regulatory processes of the cell that reduce the response of leukemic cells to treatment with anticancer drugs [17]. In our previous work, we provided evidence that the development of MDR mediated by ABCB1 overexpression in human AML cells is accompanied by alterations in several protein expressions [18,20].

The presence of latrophilin-1 has been observed in the plasma membrane of myeloid leukemia blasts from patients with AML but not in healthy mature leukocytes [26]. We also verified the presence of this protein in the AML lines MOLM-13 and SKM-1 [30]. However, we found that latrophilin-1 was downregulated in ABCB1-positive MOLM-13/VCR and SKM-1/VCR variants. In this study, we studied samples from 17 newly diagnosed patients with MDS. Interestingly, in samples positive for *ABCB1* expression (7 of 17), we also observed the presence of *ABCC1* gene expression. In the other two samples, we observed the expression of *ABCC1* alone. All nine samples expressing either *ABCB1* together with *ABCC1* or expressing *ABCC1* alone had reduced *ADGRL1* (encoding LPHN1) gene expression (more than 6-fold) compared to the seven samples without drug transporter expression (Figure 1). In one case, lower expression of *ADGRL1* was observed, although there was no expression of drug transporters. Taken together, this study showed that *ADGRL1* is another gene whose expression profile changes with the expression of *ABCB1/ABCC1* genes in malignant myeloid blasts.

LPHN1 is predominantly expressed in neurons, where it regulates neurotransmitter release by regulating calcium homeostasis [52,53,54,55]. Only a few studies have described its expression and function in other tissues, and information about its involvement in carcinogenesis is especially contradictory [29]. Goncalves Silva et al. [24] stated that LPHN1 is the regulatory molecule of galectin-9—TIM-3 signaling in AML cells. These authors observed the upregulation of GAL-9 and TIM-3 through activation of the protein kinase C (PKC)/MTOR pathway in an LPHN1-dependent manner, while activation of LPHN1 was achieved by its exogenous (LTX) and endogenous (protein FLRT3) ligands. Because we observed the downregulation of LPHN1 in drug-resistant AML cells, we hypothesized that the expression patterns of GAL-9 and TIM-3 would be altered in AML cells with developed MDR.

By qRT-PCR, we observed the upregulation of the *HAVCR2* gene encoding TIM-3 in *ABCB1*-positive cell lines. In the case of SKM-1/VCR, this occurred in a marginally significant manner, while massive and statistically significant upregulation was detected in MOLM-13/VCR cells. Increased levels of TIM-3 protein in resistant cells were subsequently confirmed by Western blotting. However, we detected several protein bands with an anti-TIM-3 specific antibody. The predicted molecular weight of TIM-3 (according to its amino acid sequence) is approximately 33 kDa; we observed a protein band at a molecular weight of approximately 35 kDa, which could be an unglycosylated TIM-3 polypeptide. This protein was upregulated in SKM-1/VCR and MOLM-13/VCR cells. The next observed protein band was approximately 50–55 kDa (which could be mature TIM-3 glycoprotein), and its expression was increased in resistant cell lines as well. Interestingly, Goncalves Silva et al. [24] observed 52 kDa bands in THP-1 cells, and they assumed that this band corresponded to the GAL-9/TIM-3 unbroken complex (because this band was detectable by both anti-TIM-3 and anti-GAL-9 antibodies). Asayama et al. [56] used several MDS cell lines for TIM-3 profiling, including SKM-1, and they observed an approximately 45 kDa band. In fact, the 33–35 kDa protein band corresponds with the predicted unglycosylated TIM-3 polypeptide; however, posttranslational modifications (particularly N-glycosylation) could lead to a higher molecular mass of Tim-3 in the 50–70 kDa range, which was observed in multiple tissues and cell types [57,58,59]. This is consistent with our data, since we observed a 70 kDa band in all cell lines used (see Supplementary files), and this fragment was upregulated in ABCB1-positive cells as well. Finally, a smaller protein band of approximately 20 kDa was detected by Western blotting (see Supplementary files), with higher expression levels in both resistant cell lines than in their parental cell lines. This protein band was also observed in THP-1 cells [24]. Smaller fragments of TIM-3 are probably the shedding products of TIM-3. These observations were also confirmed in cystic fibrosis cells, where TIM-3 was shed by serine proteases [59]. We assume that all of these protein bands correspond to TIM-3, but further proteomic analysis would clarify the posttranslational modifications and protein shedding and their impact on the function of this protein in AML cells.

Nevertheless, our results regarding increased expression of TIM-3 (all the protein bands detected) in drug-resistant cells are consistent with other works. Horlad et al. [60] proved that overexpression of TIM-3 in adult T-cell leukemia/lymphoma was associated with chemoresistance; furthermore, *HAVCR2* gene transfection directly led to chemoresistance development in ATN-1 cells. Moreover, the overexpression of TIM-3 on T cell subsets of patients with AML who failed chemotherapy is well known in clinical practice [61]. Finally, Asayama et al. [56] found higher levels of TIM-3 in patients with advanced stages of MDS. Thus, the overexpression of TIM-3 might be related to adverse prognosis, chemoresistance and/or advanced stages of certain hematopoietic disorders.

This study focused on the molecular forms of GAL-9. There is strong evidence that the GAL-9 gene has multiple isoforms due to alternative splicing of pre-mRNA [33,34,62,63,64], and we have further investigated this topic. To the best of our knowledge, this is the first report that *LGALS9* is alternatively spliced in AML cells, leading to the production of all eight transcript variants predicted from the NCBI gene database. 

We found that the individual isoforms have a different expression profile in ABCB1-positive cells than in their sensitive parental counterparts. Additionally, the resistant cell variants SKM-1/VCR and MOLM-13/VCR differed in their GAL-9 isoform profile. This could be explained by the different characteristics of the parental cell lines. Although these cell lines were derived from patients who developed AML after previous MDS, SKM-1 was derived from an elderly patient [42], while MOLM-13 was derived from a young patient in the first relapse of disease [41]. These cells are also different in terms of the occurrence of gene mutations, chromosomal aberrations, and the altered ploidy of chromosomes (reviewed in [38]). Thus, the above characteristics, including the previous medical and treatment history of the patient donors, may contribute to the observed differences. Notably, the total mRNA expression of *LGALS9* (detected by qRT-PCR) was significantly decreased in both drug-resistant cell lines compared to the parental cell lines.

The GAL-9 protein isoform Western blot results were partially consistent with those obtained from mRNA profiling. However, isoforms 3 and X3 were not found in all independent experiments, so we excluded these two isoforms from further evaluation. These isoforms may not be constitutively induced, and their expression might change dynamically during cellular homeostasis. Alternatively, they could be preferentially released into the extracellular matrix (ECM) of AML blasts and thus are difficult to detect in cells. However, we must acknowledge a degree of inconsistency when comparing cellular mRNA contents and their protein products for individual GAL-9 isoforms. For example, when comparing SKM-1/VCR- and MOLM-13/VCR-resistant cells with sensitive counterparts (SKM-1 and MOLM-13), the amount of TV1 encoding full-length GAL-9 (isoform long) was reduced, while this protein isoform was increased. These discrepancies might have various causes, such as:

1. The induction of transcription may not be the sole regulatory mechanism for GAL-9 expression in AML cells; other mechanisms may be involved. The level of translation can be significantly limited by RNA interference, e.g., microRNA-22 decreases GAL-9 levels [65]. There is considerable *ABCB1* expression in SKM-1/VCR and MOLM-13/VCR cells, and it has been shown that mRNA turnover and translation initiation may play a crucial role in its expression, not just the induction of transcription [66]. It is possible that the expression of other genes in ABCB1-positive cells may behave similarly. The protein level in cells can be controlled by ubiquitination and degradation in the proteasome. This pathway may be differentially active in ABCB1-positive and ABCB1-negative leukemia cells [67]. Different levels of proteasomal degradation of individual GAL-9 isoforms can also be expected.
2. There are lines of evidence that GAL-9 is secreted into the ECM, where it might play a role in intercellular junctions [45,68]. GAL-9 affects AML cells through an autocrine loop with TIM-3, leading to activation of survival signaling pathways and directing the ability of AML blasts to self-renew [31] and/or affect immune cells, thus participating in the mechanisms by which AML blasts escape control of the immune system [24,69].

As described by [63], alternative splicing of GAL-9 is limited to exons 5, 6 and 10. A special case is probably isoform X5, which consists of the first five exons identical to FL but contains a unique atypical exon 6′. When comparing the sequences of exon 6′ with other parts of the *LGALS9* gene, we found a similarity to intron 5–6. Therefore, we conclude that exon 6′ represents the portion of intron 5–6 that is retained in the coding sequence. However, exon 6′ contains a premature stop codon, presumably leading to truncation of the linker peptide and the absence of C-CRD. Splicing of exon 10 in isoforms 3, X3, and X4 results in a C-CRD truncation due to a frame shift and subsequent premature stop codon in exon 11. The function of these truncated isoforms remains unclear. However, the splicing of exons 5 and 6 affects the length of the linker peptide, which determines the rotational freedom of both CRDs and, consequently, the valency of GAL-9 [63,70].

At present, the reason for the existence of several GAL-9 isoforms, for which the respective TVs and corresponding proteins can be identified, is not understood. Zhang et al. [71] found that the exogenous long, short isoform and X2 isoform (alternative original names are galectin-9L, M and S) have different effects on E-selectin levels in LoVo colon carcinoma cells. Aanhane et al. [48] further demonstrated that the individual monovalent domains of N-CRD (and C-CRD of galectin-9) have the opposite effect on angiogenesis as the short isoform (GAL-9M) in an experimental model of chicken chorioallantoic membrane. GAL-9 isoform deregulation was also observed during normal pregnancy and spontaneous abortions in mouse models and in human patients [33]. Diverse functions of individual isoforms could explain the potent and multivariable immunomodulatory effects of GAL-9. For instance, in the case of monocytes, the effect of GAL-9 depends on its cell localization—intracellular proteins induce a proinflammatory phenotype of monocytes, but extracellular localization can lead to the induction of cell death [72]. This is also supported by the fact that exogenously modified human GAL-9 exhibited a proapoptotic effect on five different derived cell lines of chronic myelogenous leukemia [73], while endogenous GAL-9 was shown to drive the self-renewal capacity of AML blasts [31]. Using online prediction tools, we compared some of the posttranslational modifications of each GAL-9 isoform. Interestingly, the number of predicted N-glycosylation sites was similar for all isoforms, but there was a difference in the number of predicted O-glycosylation sites (Supplementary Table S2). Thus, it is necessary to elucidate the glycosylation profiles of the isoforms and to determine whether this modification also influences protein function or localization.

**Figure 10:**
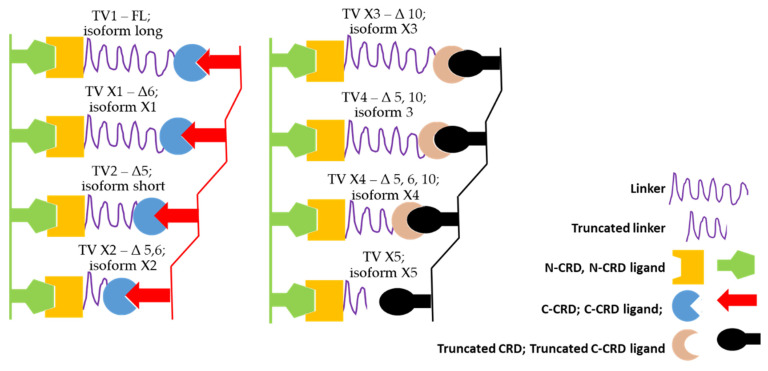
Hypothesis of galectin-9 truncation in modulating its function. Truncating the linker peptide by splicing exons 5 and/or 6 can modulate the distance at which the divalent lectin is able to link two specific sites. C-CRD truncation (splicing of exon 10) may alter the specificity of oligosugars. The protein variant X5 appears to be a monovalent lectin and can either label sites with specific ligands for further interactions or, conversely, block them before they can interact with divalent lectins, thereby preventing efficient cross-linking.

Here we suggest a simple hypothesis about the different function of individual isoforms. Alternative splicing of exons 5 and 6 affects the length of the linker peptide between N-CRD and C-CRD (for isoforms X1, isoform short and X2 with unchanged C-CRD and isoforms 3 and X4 with truncated C-CRD). This change limits the distance at which a double-valent lectin can link two sites with suitable ligands for its CRDs. Thus, isoforms with a truncated linker will be functional at shorter distances compared with the full-length protein (Figure 10).

Another situation occurs in the case of exon 10 splicing, which probably changes the specificity of this site to sugar ligands. Thus, this change may indicate that truncated C-CRD will bind to possible saccharide ligands with altered affinities. Therefore, truncated C-CRD would be expected to prefer a ligand other than that typical of the complete variant (Figure 10). Isoform X5 probably does not contain C-CRD and can specifically label cells for various other interactions. However, as a monofunctional variant, it could act as an antagonist and, upon binding of its N-CRD to a suitable saccharide ligand, block it from interacting with the bifunctional variants and prevent the formation of a linkage between two specific sites. GAL-9 ligands can be bivalent (a linear linkage is produced), trivalent or tetravalent (typical networks are produced) [35]. Complex interconnected structures are formed with the participation of bi-, tri- and tetravalent carbohydrates. The various types of GAL-9 isoforms, together with the differently structured oligosaccharide portions of the glycoproteins, will then structure these networks. The saccharide ligands of GAL-9 have a relatively complex structure (they are composed of mannose, galactose, fucose, N-acetylglucosamine and N-acetylgalactosamine). Branched N-glycans (9–14 subunits) bind to both N-CRD and C-CRD, some of which bind simultaneously to both GAL-9 CRDs, increasing the affinity of the interaction [35]. Relatively simpler penta- and hexasaccharides are distinguished and bound only by N-CRD.

Finally, we investigated the expression profile of CD44 in the AML cell lines SKM-1 and MOLM-13 and their drug-resistant counterparts, which overexpress the ABCB1 protein. We undertook this investigation because it was previously indicated that CD44 is another receptor/binding partner of GAL-9 [35,44,45,46]. Furthermore, it has been described that high expression levels of certain CD44 isoforms correlates with shorter survival of AML patients [74] and risk of relapse [75].

At the mRNA level, we observed *CD44* upregulation only in SKM-1/VCR cells in comparison to the sensitive parental cell line, but only in a marginally significant manner (Figure 2). In the case of MOLM-13, *CD44* transcript was detected at a comparable level to that in the drug-resistant counterpart. Interestingly, the differences between drug-resistant and drug-sensitive cell lines were detected by Western blotting for both cell lines. Such a discrepancy was also observed by [75] in mouse xenograft models of AML, where the *CD44* mRNA levels remained unchanged, but the protein level on the plasma membrane was elevated. We identified three protein bands (38, 46 and 85–95 kDa) when using an anti-CD44-specific antibody (Figure 4). Massive upregulation of the highest molecular weight band was observed in both drug-resistant cell lines compared with that in the parental cell lines. CD44 has multiple isoforms because of alternative splicing, while the 85–95 kDa protein corresponds to the standard isoform (CD44s). Our results are consistent with data from other groups [74,76], since CD44s is, together with CD44v3-v10 (retention of exons 7–14 or v3–v10, while exon 18 is spliced out) and CD44v6 (retention of exon 10 or v6), most typical for AML (reviewed in [77,78]). The CD44s isoform is a protein product of a transcript variant in which all variable exons (exons 6–14 or v2–v10) are spliced out. The molecular weight of an unmodified protein is approximately 38 kDa, while massive glycosylation leads to an increase in molecular weight to almost 95 kDa. We assume that the 38 kDa band detected by Western blotting could in fact be the deglycosylated form of CD44s. Furthermore, the 46 kDa band could represent isoform CD44v10 (retention of exon 14 or v10, splicing of exon 18), whose molecular weight of the deglycosylated form is exactly 46 kDa (again, massive glycosylation leads to a total MW of 120 kDa; [79]). However, alternative splicing was not the object of this study, and further analysis is needed.

The observed upregulation of CD44s in drug-resistant, ABCB1-positive AML cells is also consistent with other groups in relation to chemoresistance development. Wang et al. [80] concluded that chemoresistance of the HL60 cell line to adriamycin (ADM) and cytarabine (Ara-C) was mediated by CD44, since knockdown of this protein led to reversion of chemoresistance and sensitization of HL60 cells to ADM and Ara-C. A correlation between CD44 expression and chemoresistance was also reported in T-cell acute lymphoblastic leukemia. Interestingly, CD44 upregulation was also observed in residual blasts after induction chemotherapy when compared to matched pretreatment samples [81]. CD44 involvement in chemoresistance was confirmed in non-small-cell lung cancer cells [82] and head and neck squamous cell carcinoma [83]. Thus, it is possible that CD44 itself contributes to chemoresistance development.

## 5. Conclusions

Latrophilin-1 is found in leukemic myeloid blasts but not in healthy leukocytes [26]. In a previous study, we showed that overexpression of ABCB1 leads to decreased latrophilin-1 expression in SKM-1 and MOLM-13 cells [30]. In the present work, we demonstrated such a reciprocal relationship between ABCB1 and latrophilin-1 expression in samples of patients newly diagnosed with myelodysplastic syndrome without prior treatment. Based on a comparison of ABCB1-positive MOLM-13/VCRs and SKM-1/VCRs with their ABCB1-negative MOLM-13 and SKM-1 counterparts, we found that ABCB1 overexpression results in i) upregulation of TIM-3 (at both the mRNA and protein levels of the 35 and 55 kDa forms); and ii) downregulation of GAL-9 (at the mRNA level, the protein level could not be quantified due to the existence of multiple protein isoforms). We identified eight transcriptional variants of the *LGALS9* gene (encoding GAL-9) and verified their structure by sequencing. In addition, for each transcriptional variant, we observed its protein product with a typical molecular weight. The protein profile of these isoforms differs in ABCB1-positive cells (MOLM-13/VCR and SKM-1/VCR) and ABCB1-negative cells (MOLM-13 and SKM-1). Both TIM-3 and GAL-9 may allow myeloid blasts to escape from the immune system after initiation by latrophilin-1 [24]. The expression of *CD44* (another receptor/binding partner of GAL-9 [44,46]) was slightly increased at the mRNA level in SKM-1/VCR variants compared to the original SKM-1 cells. Accordingly, we found an increase in the 85–95 kDa isoform of CD44 observed in SKM-1/VCR cells. There was no such significant increase in MOLM-13/VCR; in contrast, we observed a significant decrease in the level of the smallest 38 kDa form.

## Figures and Tables

**Table 1:** Overview of TVs and isoforms of GAL-9 with NCBI gene database ID.

Alternative Splicing	Transcript Variant Name and ID	Protein Isoform Name and ID
Full-length (FL)	TV1; NM_009587.3	Isoform long; NP_033665.1
Δ6	X1; XM_017024623.2	Isoform X1; XP_016880112.1
Δ5	TV2; NM_002308.4	Isoform short; NP_002299.2
Δ5,6	X2; XM_006721892.3	Isoform X2; XP_006721955.1
Δ10	X3; XM_006721893.3	Isoform X3; XP_006721956.1
Δ5,10	TV4; NM_001330163.2	Isoform 3; NP_001317092.1
Δ5,6,10	X4; XM_006721895.4	Isoform X4; XP_006721958.1
Exon 6′; premature stop	X5; 011524796.2	Isoform X5; XP_011523098.1

## Data Availability

Additional data and the resistant variants of MOLM-13 and SKM-1 cells are available from the authors.

## Supplementary Materials

The following are available online at https://www.mdpi.com/article/10.3390/cancers13143629/s1: Table S1: PCR primers used in this study, with T_A_ and respective PCR products, Table S2: Comparison of N-glycosylation and O-glycosylation site prediction of GAL-9 isoforms, Table S3: Sequencing identification details for *LGALS9* TV amplicons, Table S4: Summary of gene and protein expression in resistant SKM-1/VCR cells compared to parental sensitive variant SKM-1, Table S5: Summary of gene and protein expression in resistant MOLM-13/VCR cells compared to parental sensitive variant MOLM-13, Figure S1: Design of primers for RT-PCR detection of *LGALS9* splice variants, Figure S2: Representative agarose gel of *ADGRL1, ABCB1* and *ABCC1* detection in MDS clinical samples, Figure S3: Expression of *ABCB1* and *ABCC1* in SKM-1 and MOLM-13 and their resistant counterparts, Figure S4: Full alignment of Intron 5–6 from FL *LGALS9* and exon 6′ from X5 variant, Figure S5: Further characterization of splice variant X5 of GAL-9, Figure S6: Uncropped agarose gel of *ADGRL1* detection (Figure S2), Figure S7: Uncropped agarose gel of *ABCB1* detection (Figure S2), Figure S8: Uncropped agarose gel of *ABCC1* detection (Figure S2), Figure S9: Uncropped agarose gel of *ACTB* detection (Figure S2), Figure S10: Uncropped agarose gel of *LGALS9, HAVCR2* and *CD44* detection (Figure 2), Figure S11: Uncropped membrane of TIM-3 and GAPDH protein detection by Western blotting (Figure 3), Figure S12: Densitometric analysis of 70 kDa and 20 kDa protein forms of TIM-3 detected by Western blot, Figure S13: Uncropped membrane of CD44 protein detection by Western blotting (Figure 4), Figure S14: Uncropped membrane of GAPDH protein detection by Western blotting (Figure 4), Figure S15: Uncropped agarose gel of TVs of *LGALS9* detection (Figure 8), Figure S16: Uncropped agarose gel of *LGALS9*—X5 detection (Figure 8), Figure S17: Uncropped agarose gel of *ACTB* detection (Figure 8), Figure S18: Uncropped membrane of GAL-9 and GAPDH protein detection by Western blotting (Figure 9), Figure S19: Uncropped agarose gel of *ABCC1* detection (Figure S3), Figure S20: Uncropped agarose gel of *ABCB1* detection (Figure S3), Figure S21: Uncropped membrane of ABCB1 and GAPDH detection by Western Blot (Figure S3).
