# Supplementary material for: Development of Multidrug Resistance in Acute Myeloid Leukemia Is Associated with Alterations of the LPHN1/GAL-9/TIM-3 Signaling Pathway
Source: Cancers (Basel). 2021 Jul 20;13(14):3629. doi: 10.3390/cancers13143629 (PMC8304048; doi:10.3390/cancers13143629)
Supplement: Supplementary file 1 [file cancers-13-03629-s001.zip › Supplementary files 2.pdf]

ADGRL1

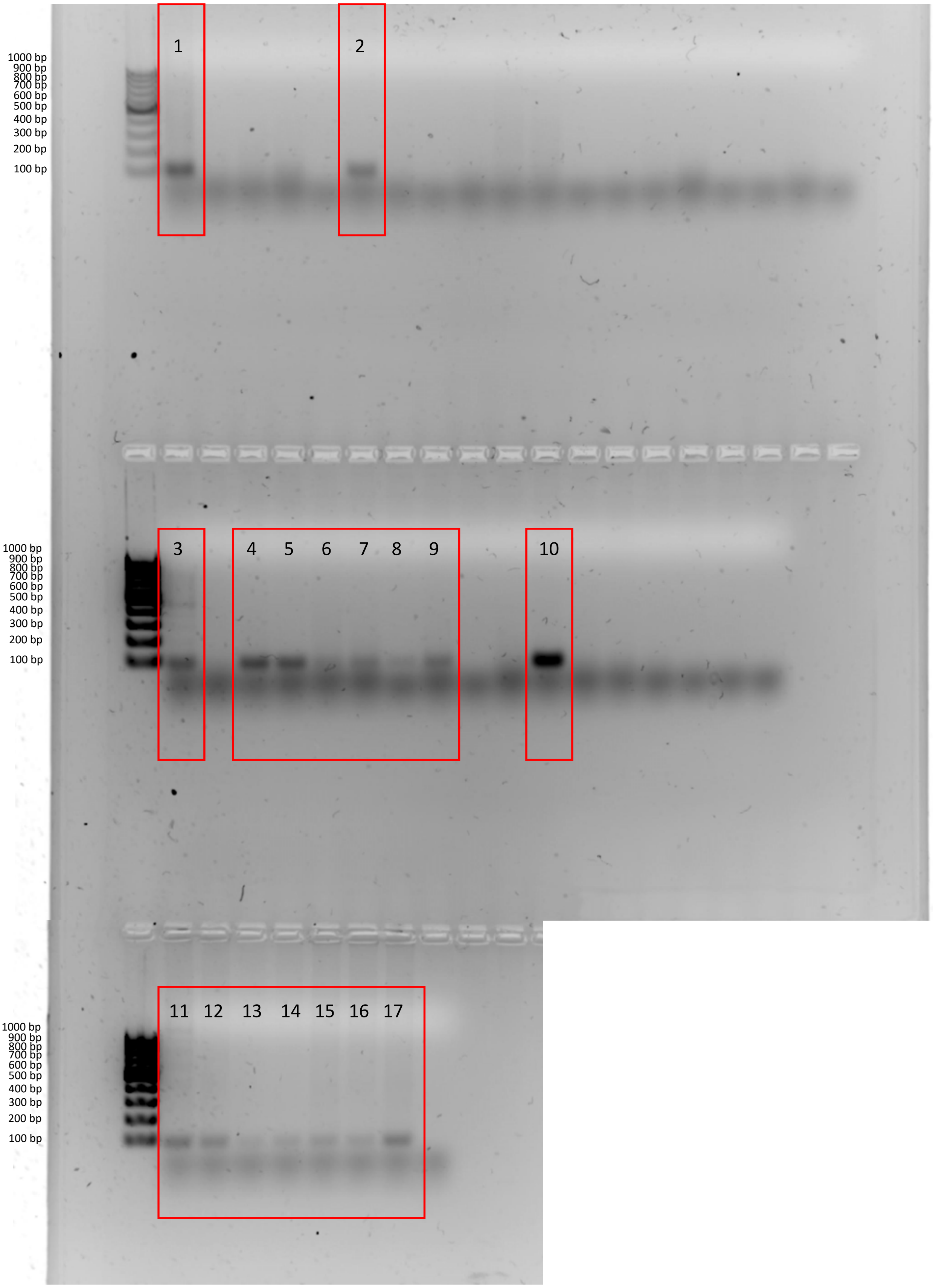

Figure S6: Uncropped agarose gel of *ADGRL1* detection (Figure S2) by RT-PCR and subsequent electroforetic separation of PCR products from primary MDS cases (1-17). Study of *ADGRL1* expression was double blinded, we worked with several cases without knowing the diagnosis of individual cases at the time of experimental procedures. Diagnosis was revealed after the experiments. 17 MDS cases studied are highlighted in red frames.

ABCB1

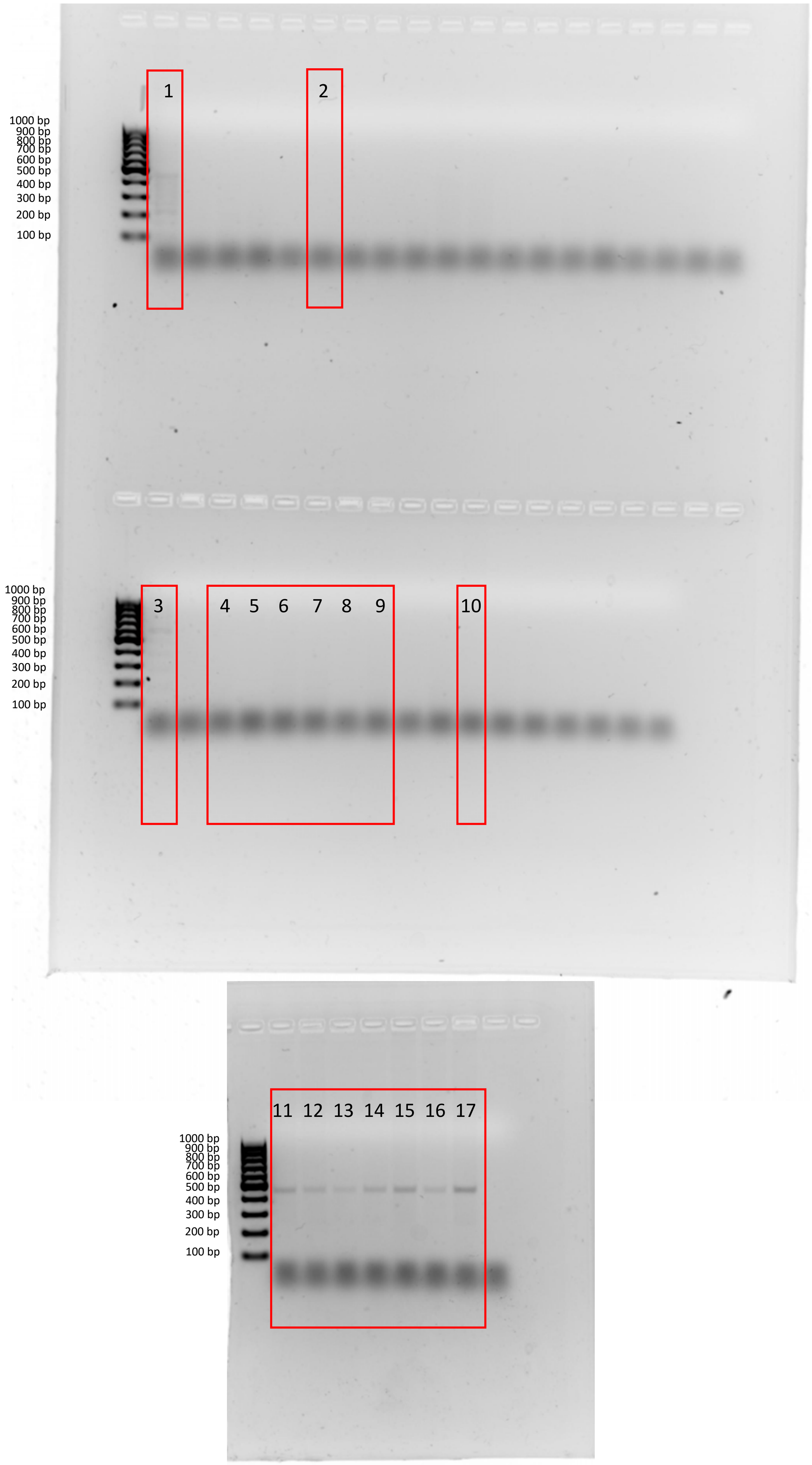

Figure S7: Uncropped agarose gel of *ABCB1* detection (Figure S2) by RT-PCR and subsequent electroforetic separation of PCR products from primary MDS cases

ABCC1

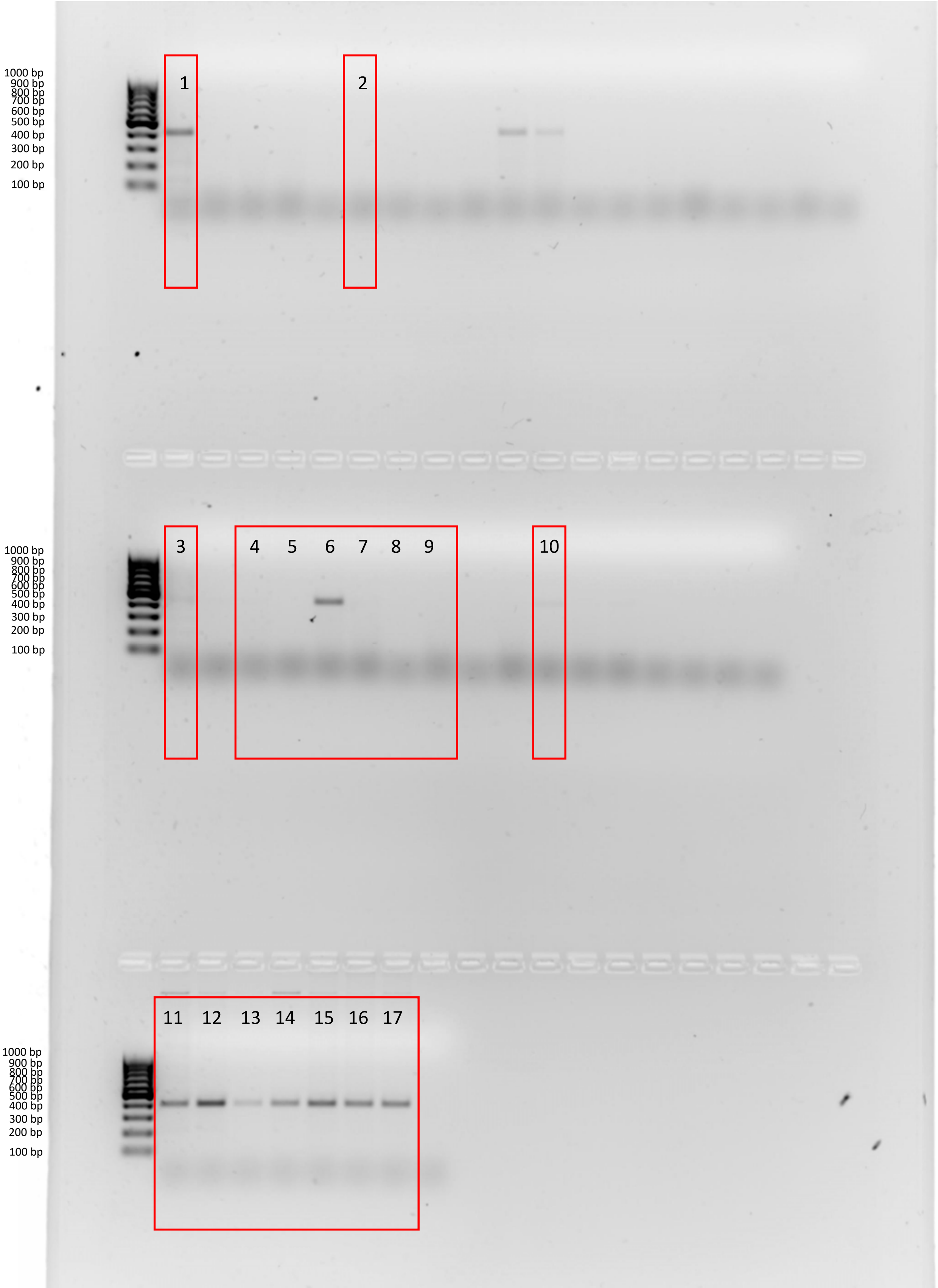

Figure S8: Uncropped agarose gel of *ABCC1* detection (Figure S2) by RT-PCR and subsequent electroforetic separation of PCR products from primary MDS cases

ACTB

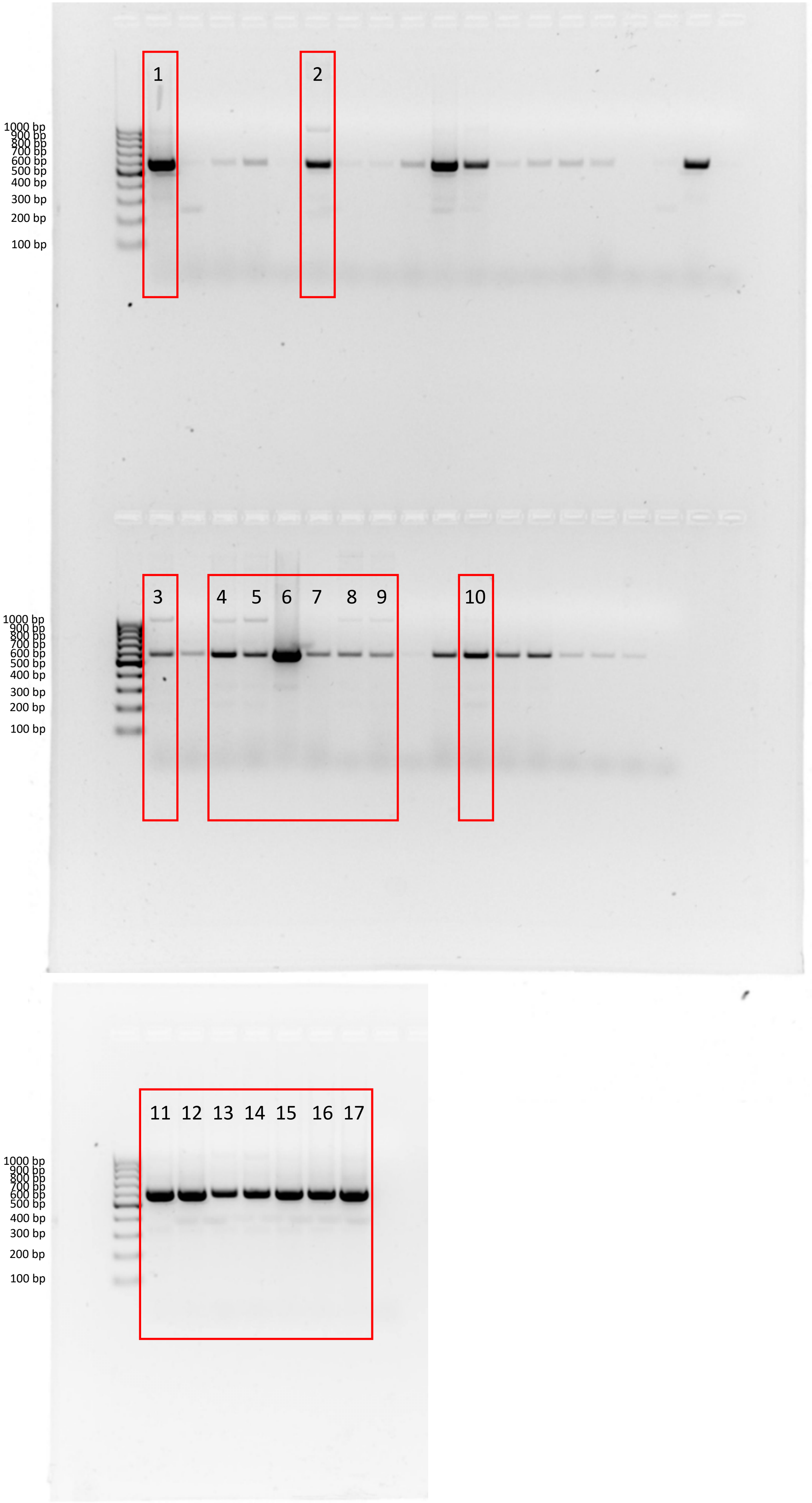

Figure S9: Uncropped agarose gel of *ACTB* detection (Figure S2) by RT-PCR and subsequent electroforetic separation of PCR products from primary MDS cases

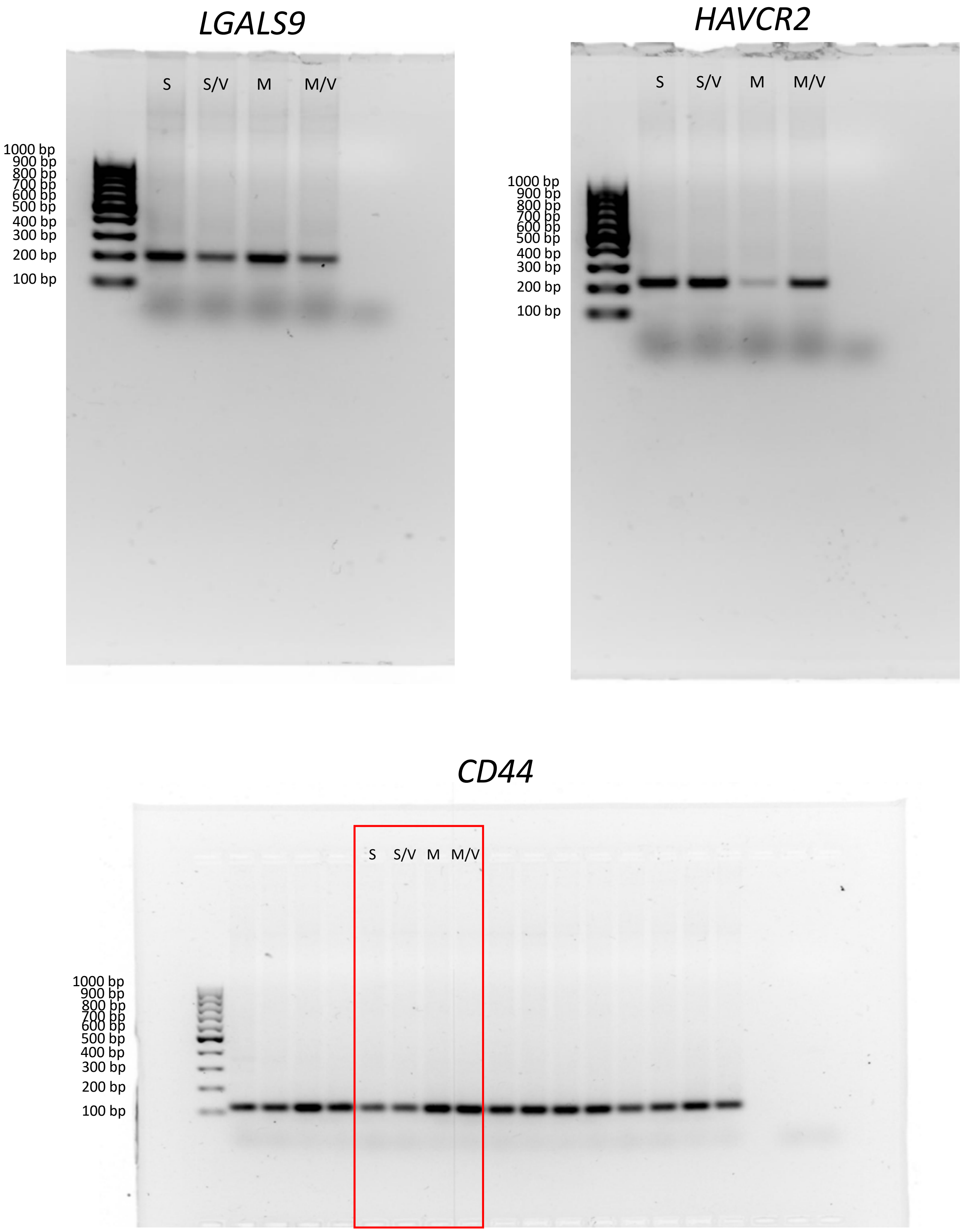

Figure S10: Uncropped agarose gel of *LGALS9, HAVCR2* and *CD44* detection (Figure 2) by RT-PCR and subsequent electroforetic separation of PCR products

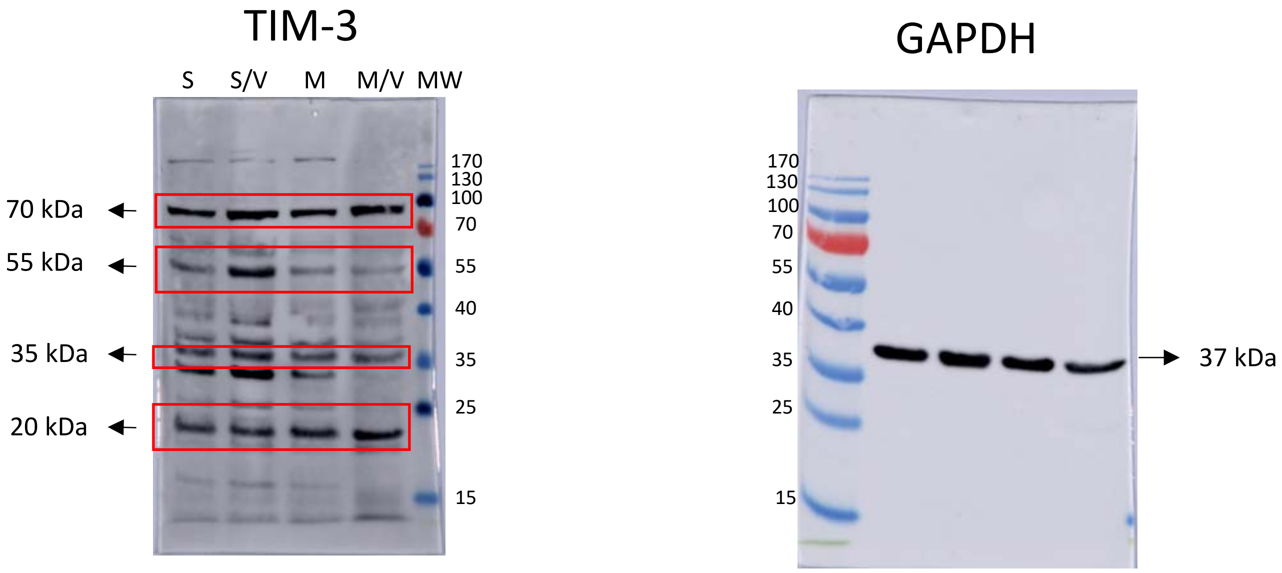

Figure S11: Uncropped membrane of TIM-3 (left panel) and GAPDH (right panel) protein detection by Western blotting (Figure 3). MW – marker of molecular weight (kDa); S – SKM-1; S/V – SKM-1/VCR; M – MOLM-13; M/V – MOLM-13/VCR. Only bands with molecular weight 70 kDa, 55 kDa, 35 kDa and 20 kDa were analysed in this study, since there is a strong evidence that abovementioned bands correspond to protein forms of TIM-3. For identification of other bands detected, further proteomical analysis is needed.

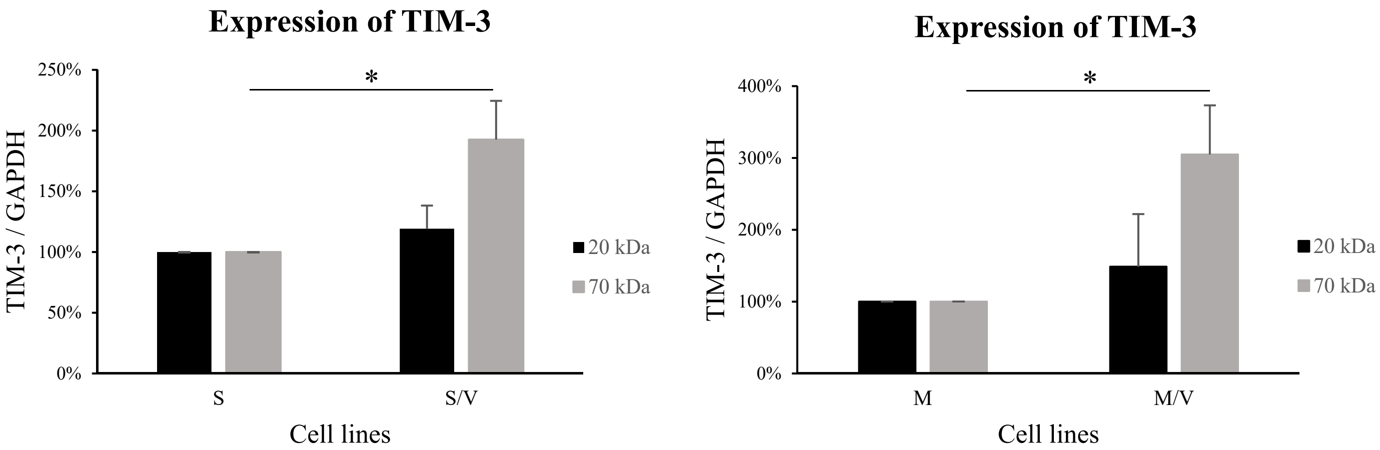

Figure S12: Densitometric analysis of 70 kDa and 20 kDa protein forms of TIM-3 detected by Western blot. Data represents mean ± SEM of 3 independent experiments. Statistical significance as follows: * $p \leq 0.05$. S – SKM-1; S/V – SKM-1/VCR; M – MOLM-13; M/V – MOLM-13/VCR.

CD44

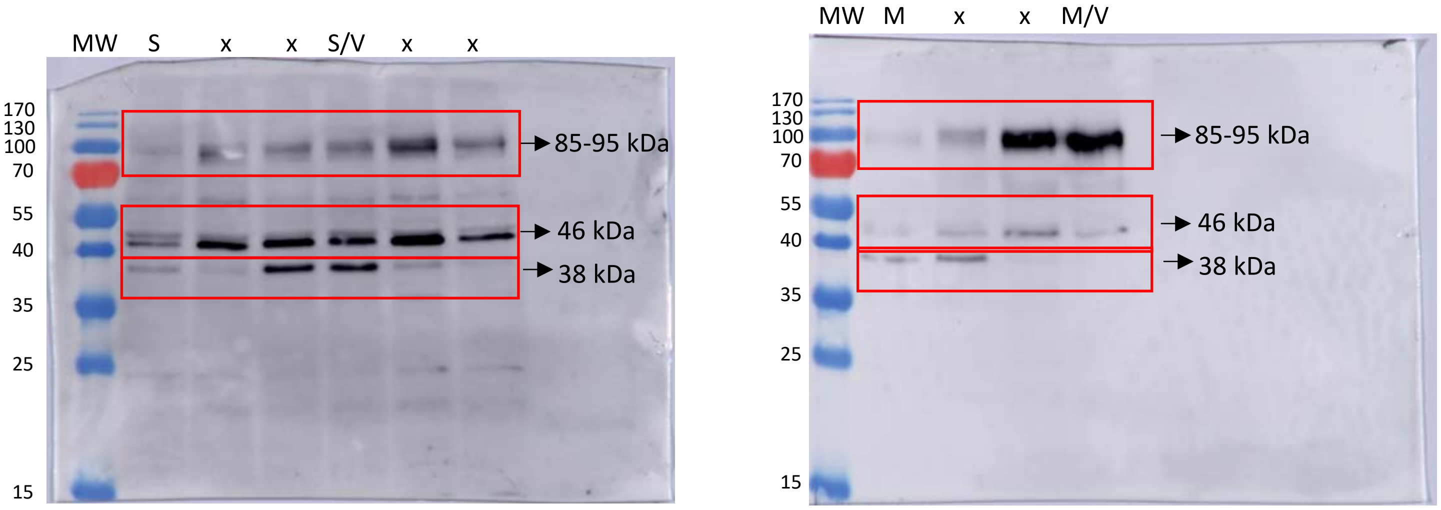

Figure S13: Uncropped membrane of CD44 protein detection by Western blotting (Figure 4). MW – marker of molecular weight (kDa); S – SKM-1; S/V – SKM-1/VCR; M – MOLM-13; M/V – MOLM-13/VCR. X - samples, that were not object of current study and were not included in any analysis.

GAPDH

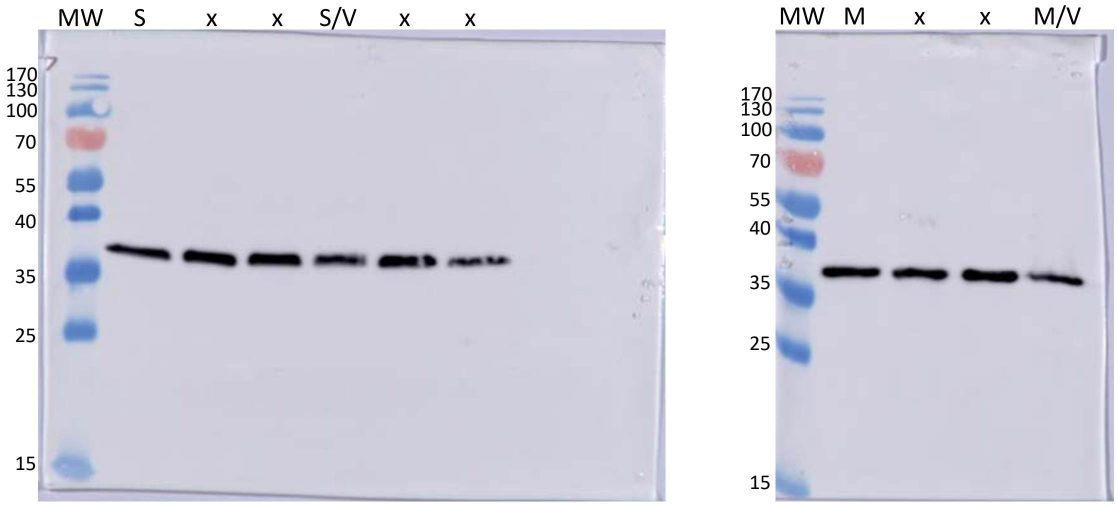

Figure S14: Uncropped membrane of GAPDH protein detection by Western blotting (Figure 4). MW – marker of molecular weight (kDa); S – SKM-1; S/V – SKM-1/VCR; M – MOLM-13; M/V – MOLM-13/VCR. X - samples, that were not object of current study and were not included in any analysis.

LGALS9 – 7 TVS

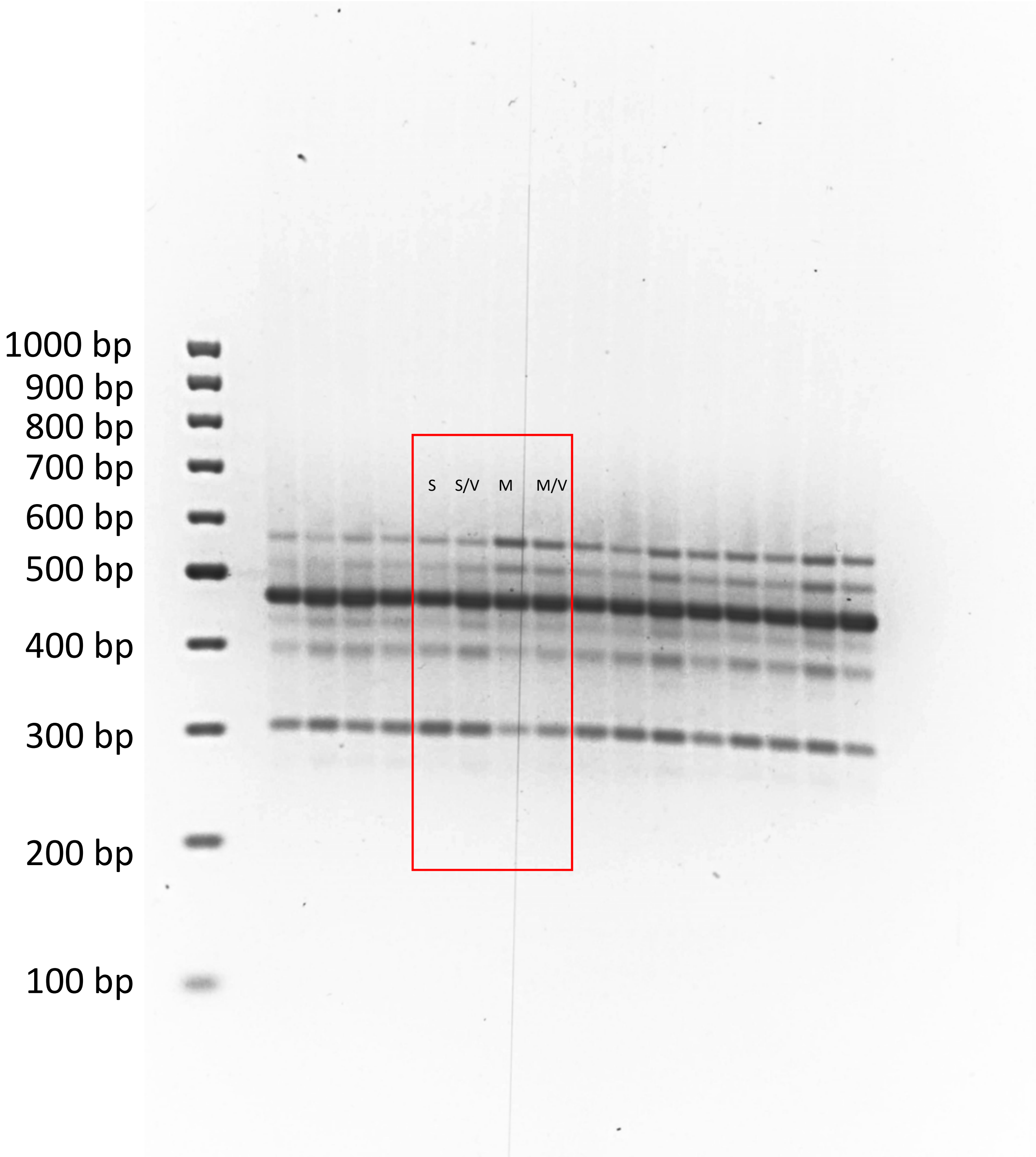

Figure S15: Uncropped agarose gel of TVs of *LGALS9* detection (Figure 8) by RT-PCR with subsequent electrophoretic separation of PCR products

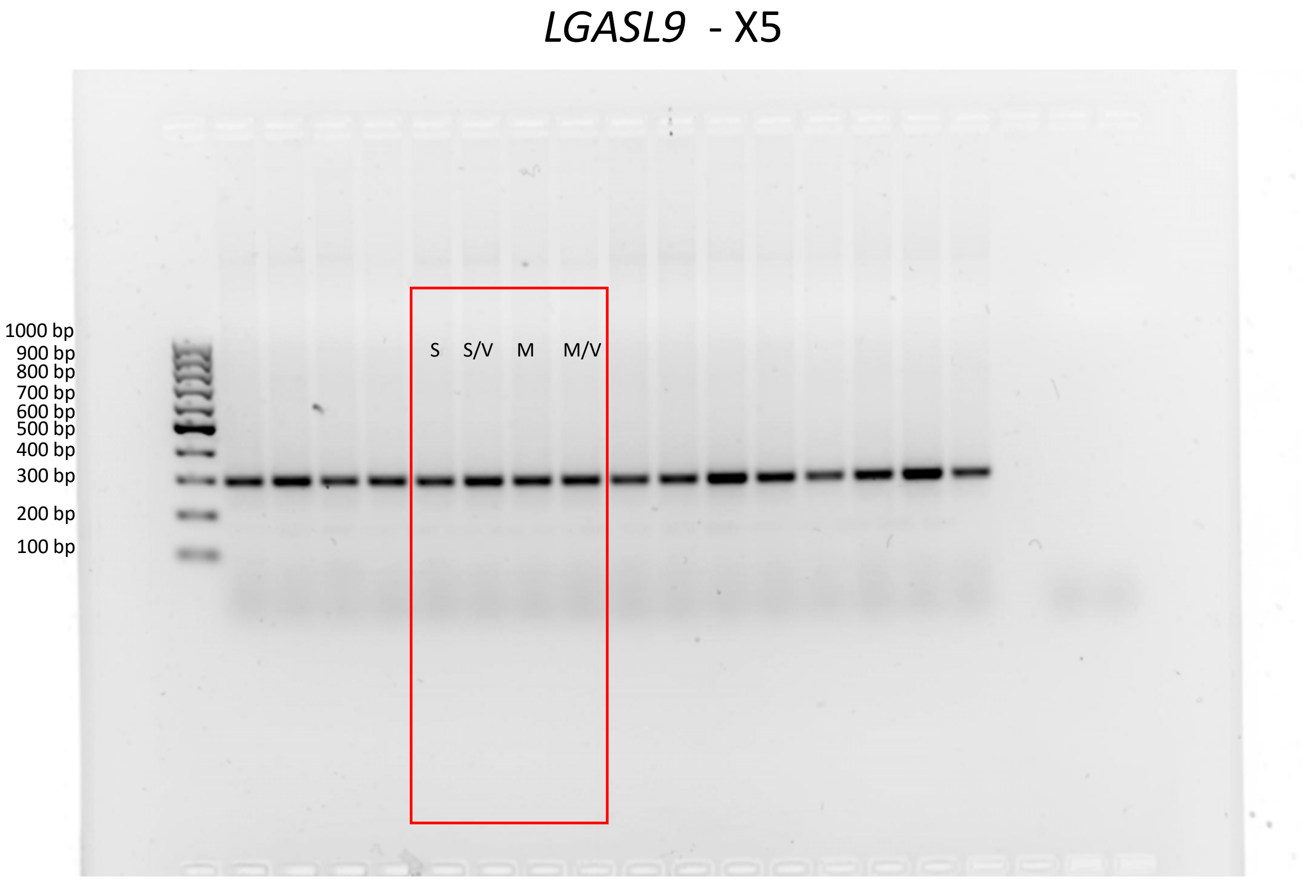

Figure S16: Uncropped agarose gel of *LGALS9 – X5* detection (Figure 8) by RT-PCR and subsequent electroforetic separation of PCR products

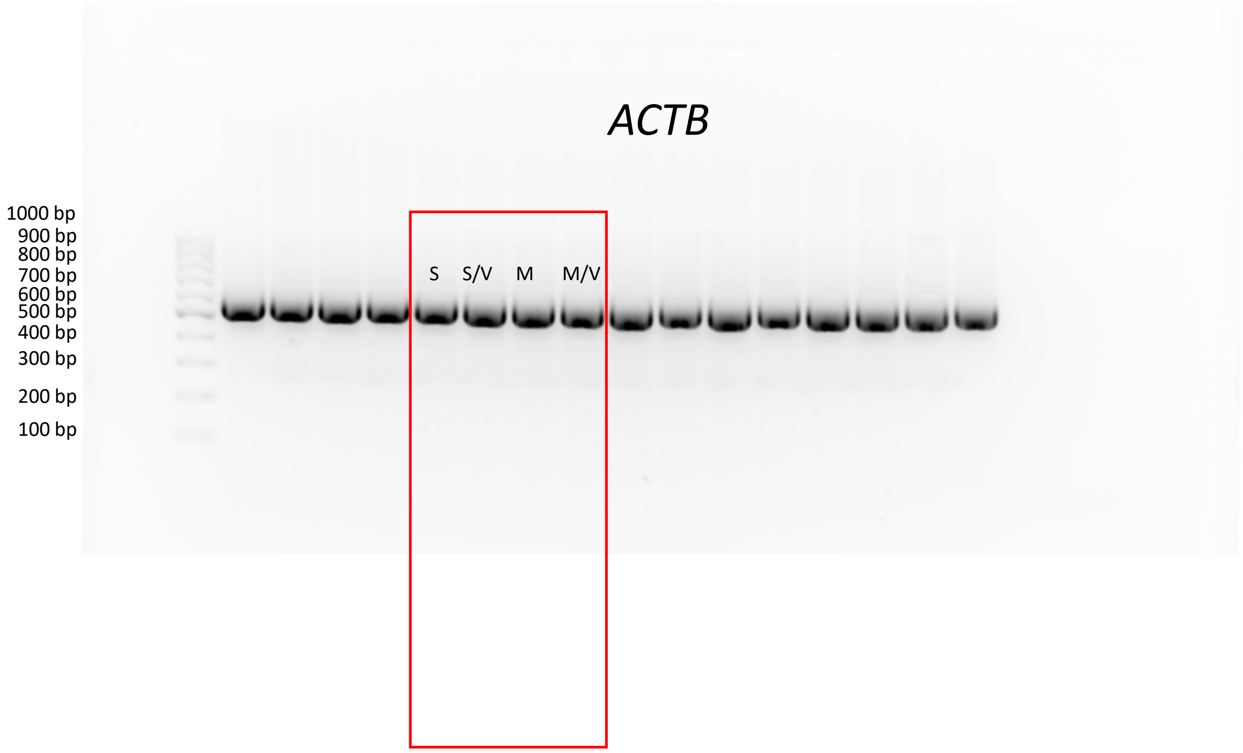

Figure S17: Uncropped agarose gel of *ACTB* detection (Figure 8) by RT-PCR and subsequent electroforetic separation of PCR products

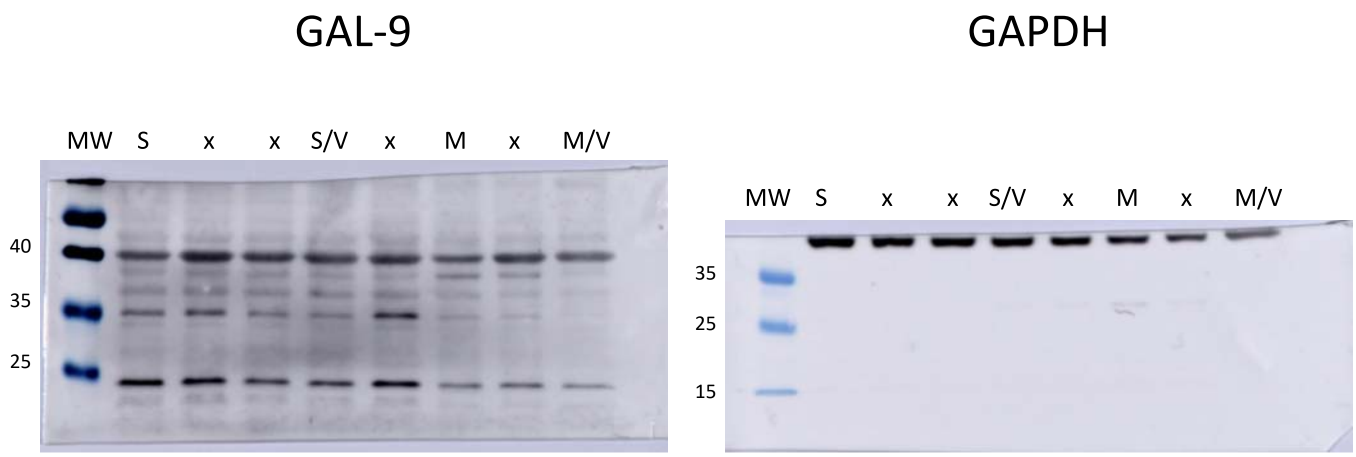

Figure S18: Uncropped membrane of GAL-9 (left panel) and GAPDH (right panel) protein detection by Western blotting (Figure 9). Individual bands represent isoforms of GAL-9. MW – marker of molecular weight (kDa); S – SKM-1; S/V – SKM-1/VCR; M – MOLM-13; M/V – MOLM-13/VCR. X - samples, that were not object of current study and were not included in any analysis.

ABCC1

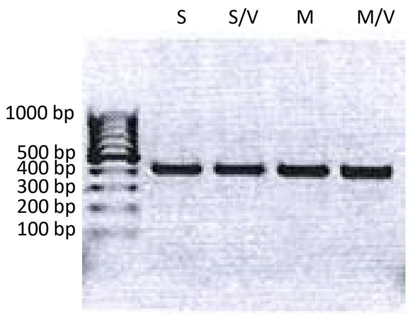

Figure S19: Uncropped agarose gel of *ABCC1* detection (Figure S3) by RT-PCR and subsequent electroforetic separation of PCR products

ABCB1

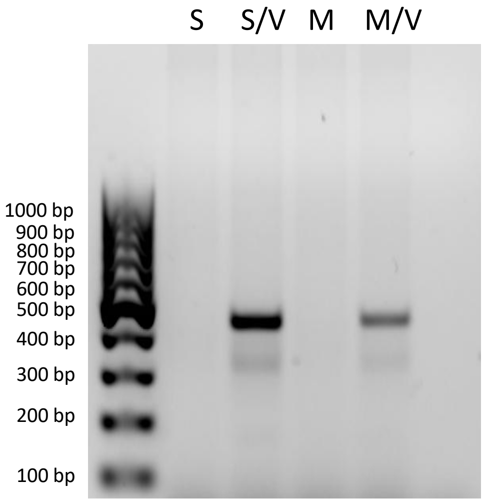

Figure S20: Uncropped agarose gel of *ABCB1* detection (Figure S3) by RT-PCR and subsequent electroforetic separation of PCR products

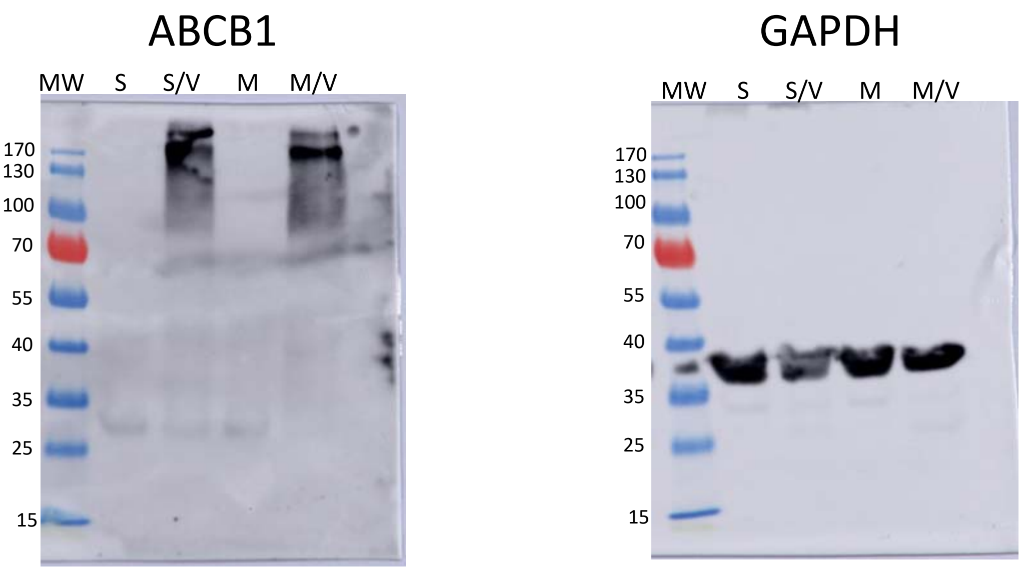

Figure S21: Uncropped membranes of ABCB1 and GAPDH detection by Western blot (Figure S3). MW – marker of molecular weitght (kDa); S – SKM-1; S/V – SKM-1; M – MOLM-13; M/V – MOLM-13/VCR
