# Supplementary material for: Development of Multidrug Resistance in Acute Myeloid Leukemia Is Associated with Alterations of the LPHN1/GAL-9/TIM-3 Signaling Pathway
Source: Cancers (Basel). 2021 Jul 20;13(14):3629. doi: 10.3390/cancers13143629 (PMC8304048; doi:10.3390/cancers13143629)
Supplement: Supplementary file 1 [file cancers-13-03629-s001.zip › Supplementary files Kocibalova a kol 2021.pdf]

Table S1. PCR primers with characterization of T_A and respective PCR products used in the study.

Target gene	Primer's sequence	T_A (°C)	PCR product (bp)
ABCB1	F: 5′-AAGTTGTATATGGTGGGAACT-3′<br>R: 5′-AATTTTGTCACCAATTCCTTCATT-3′	57.0	429
ABCC1	F: 5′-AGAAGTCTGGACGTCCCTG-3′<br>R: 5′-ACACCAAGCCGGCGTCTTT-3′	59.1	404
ACTB	F: 5′-CTGGGACGACATGGAGAAAA-3′<br>R: 5′-AAGGAAGGCTGGAGAGTCC-3′	54.4	564
ADGRL1	F: 5′-ACCTCGACACACGAGTCAAGA-3′<br>R: 5′-GATCCAGGGCATCACGTAGA-3′	56.9	90
CD44	F: 5′-CTGCCGCTTTGCAGGTGTA-3′<br>R: 5′-CATTGTGGGCAAGGTGCTATT-3′	56.0	109
HAVCR2	F: 5′-GGAATACAGAGCGGAGGTCG-3′<br>R: 5′-AGGGACACATCTCCTTTGCG-3′	56.2	213
LGALS9	F: 5′-GAAATGACATTGCCTTCCACTTCA-3′<br>R: 5′-GAAGAGGATCCCGTTCACCA-3′	56.2	194

Table S2: Comparison of N-glycosylation and O-glycosylation sites prediction of GAL-9 isoforms

GAL-9 isoform	N-glycosylation sites	O-glycosylation sites
FL; isoform long	3	8
Isoform X1	3	11
Isoform short	3	7
Isoform X2	3	5
Isoform X3	3	9
Isoform 3	3	8
Isoform X4	3	5
Isoform X5	3	8

Glycosylation sites were predicted by NetNGlyc 1.0 Server (http://www.cbs.dtu.dk/services/NetNGlyc/; Gupta R, Brunak S. Prediction of glycosylation across the human proteome and the correlation to protein function. *Pac Symp Biocomput*. 2002;:310-22. PMID: 11928486y) and NetOGlyc 4.0 Server (http://www.cbs.dtu.dk/services/NetOGlyc/ ; Steentoft C, Vakhrushev SY, Joshi HJ, Kong Y, Vester-Christensen MB, Schjoldager KT, Lavrsen K, Dabelsteen S, Pedersen NB, Marcos-Silva L, Gupta R, Bennett EP, Mandel U, Brunak S, Wandall HH, Levery SB, Clausen H.Precision mapping of the human O-GalNAc glycoproteome through SimpleCell technology. *EMBO J*, 32(10):1478-88, May 15, 2013.(doi: 10.1038/emboj.2013.79. Epub 2013 Apr 12)

Table S3: Sequencing identification details for *LGALS9* TVs amplicons

	**PCR product (bp)**	**Number of bp from sequencing / identities by BLASTN**
TV1, Full length (FL)	560	516 / 99%
X1; Δ6	524 (230)*	185 / 98%
TV2; Δ5	464	427 / 99%
X2; Δ5,6	428	427 / 99%
X3; Δ10	397 (278)*	242 / 100%
TV4; Δ5,10	301	266 / 99%
X4; Δ5,6,10	265	265 / 99%
X5; Exon 6'; Δ6-11	288	243 / 99%

* for sequencing analysis of these splice variants, special pair of primers (for each variant separatelly and specific only for X1 and X3) were used in additional experiments (data not shown) because of purification problems from agarose gel (X1 and X3 variants formed heteromers with other variants, namely TV4 and X2 and even extensive gel electrophoresis was not sufficient for separating them)

Table S4: Summary of gene and protein expression in resistant SKM-1/VCR cells compared to parental sensitive variant SKM-1

<table>
<tr><th colspan="6">SKM-1/VCR</th></tr>
<tr><th colspan="3">LGALS9/GAL-9</th><th colspan="3">HAVCR2/TIM-3</th></tr>
<tr><th>TV/isoform</th><th>mRNA</th><th>Protein</th><th>Isoform</th><th>mRNA*</th><th>Protein</th></tr>
<tr><td>TV1/isoform long</td><td>↓ (significant)</td><td>↑ (significant)</td><td>35 kDa</td><td rowspan="2">↑ (marginally significant)</td><td>↑ (significant)</td></tr>
<tr><td>X1/X1</td><td>= (ns)</td><td>↓ (ns)</td><td>55 kDa</td><td>↑ (ns)</td></tr>
<tr><td>TV2/isoform short</td><td>↑ (significant)</td><td>=</td><th colspan="3" rowspan="2">CD44/CD44</th></tr>
<tr><td>X2/X2</td><td>↑ (ns)</td><td>↓ (ns)</td></tr>
<tr><td>X3/X3</td><td>↑ (ns)</td><td>undetermined</td><th>Isoform</th><th>mRNA*</th><th>Protein</th></tr>
<tr><td>TV4/isoform 3</td><td>=</td><td>undetermined</td><td>38 kDa</td><td rowspan="3">↑ (marginally significant)</td><td>↑ (ns)</td></tr>
<tr><td>X4/X4</td><td>↑ (ns)</td><td>↓ (ns)</td><td>46 kDa</td><td>=</td></tr>
<tr><td>X5/X5</td><td>↑ (significant)</td><td>=</td><td>85-95 kDa</td><td>↑ (significant)</td></tr>
</table>

* in case of *HAVCR2* and *CD44* expression at mRNA, total level of all transcripts was determined. ↑ -upregulation; ↓ - downregulation; ns – non-significant

Table S5: Summary of gene and protein expression in resistant MOLM-13/VCR cells compared to parental sensitive variant MOLM-13

<table>
<tr><th colspan="6">MOLM-13/VCR</th></tr>
<tr><th colspan="3">LGALS9/GAL-9</th><th colspan="3">HAVCR2/TIM-3</th></tr>
<tr><th>TV/isoform</th><th>mRNA</th><th>Protein</th><th>Isoform</th><th>mRNA*</th><th>Protein</th></tr>
<tr><td>TV1/isoform long</td><td>↓ (significant)</td><td>↑ (ns)</td><td>35 kDa</td><td rowspan="2">↑ (significant)</td><td>↑ (ns)</td></tr>
<tr><td>X1/X1</td><td>↓ (significant)</td><td>↓ (significant)</td><td>55 kDa</td><td>↑ (ns)</td></tr>
<tr><td>TV2/isoform short</td><td>=</td><td>↓ (significant)</td><th colspan="3" rowspan="2">CD44/CD44</th></tr>
<tr><td>X2/X2</td><td>=</td><td>↓ (significant)</td></tr>
<tr><td>X3/X3</td><td>↓ (ns)</td><td>undetermined</td><th>Isoform</th><th>mRNA*</th><th>Protein</th></tr>
<tr><td>TV4/isoform 3</td><td>=</td><td>undetermined</td><td>38 kDa</td><td rowspan="3">=</td><td>↓ (significant)</td></tr>
<tr><td>X4/X4</td><td>↓ (ns)</td><td>↓ (ns)</td><td>46 kDa</td><td>↑ (ns)</td></tr>
<tr><td>X5/X5</td><td>=</td><td>=</td><td>85-95 kDa</td><td>↑ (ns)</td></tr>
</table>

* in case of *HAVCR2* and *CD44* expression at mRNA, total level of all transcripts was determined. ↑ -upregulation; ↓ - downregulation; ns – non-significant

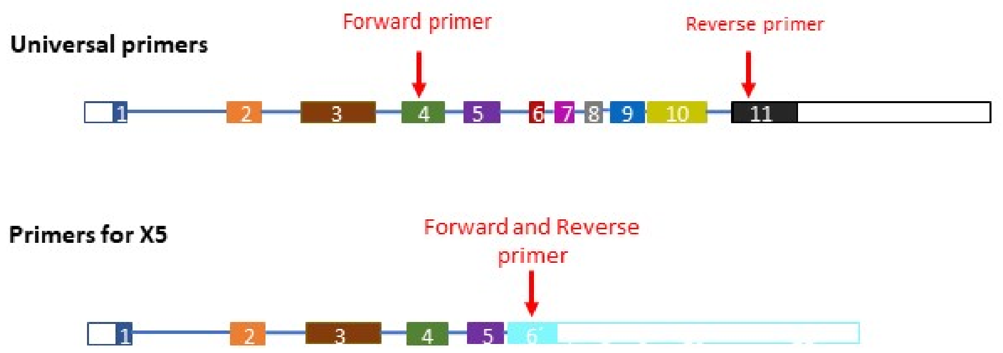

Figure S1: Design of primers for RT-PCR detection of *LGALS9* splice variants. Universal pair of primers were designed to exon 4 (forward) and 11 (reverse), exons included in 7 variants (except X5 variant). RT-PCR produced mixture of amplicons with different molecular mass. For detection of X5 variant, both primers were designed to specific exon 6´ which does not occur in other variants.

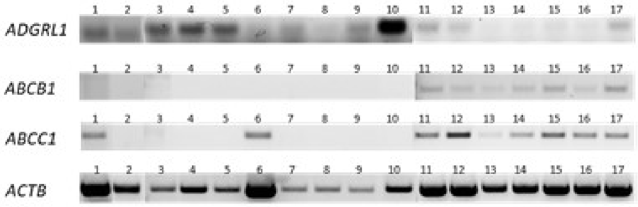

Figure S2: Representative agarose gel of *ADGRL1*, *ABCB1* and *ABCC1* detection in MDS clinical samples, where *ACTB* was used as internal control. Densitometric analysis was performed using ImageJ software.

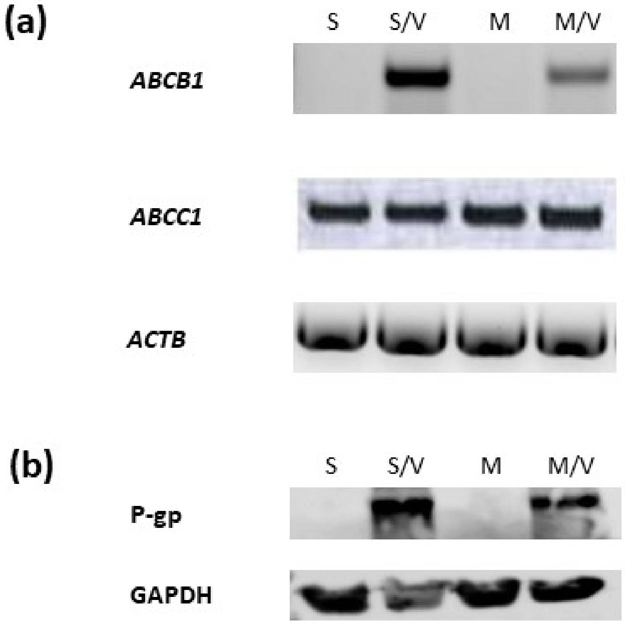

Figure S3: Expression of *ABCB1* and *ABCC1* in SKM-1 and MOLM-13 and their resistant counterparts. (a) representative agarose gel of PCR products for both genes, when *ACTB* was used as internal loading control. No changes were detected for *ABCC1* gene in drug-sensitive and respective drug-resistant variants. Upregulation of *ABCB1* as a result of selection/adaptation to VCR was detected only in SKM-1/VCR and MOLM-13/VCR, what is consistent with qRT-PCR and our previous papers. (b) representative Western blot of P-gp (product of *ABCB1* gene), whose expression was detected only in drug-resistant cell lines. S – SKM-1; S/V – SKM-1/VCR; M – MOLM-13; M/V – MOLM-13/VCR.

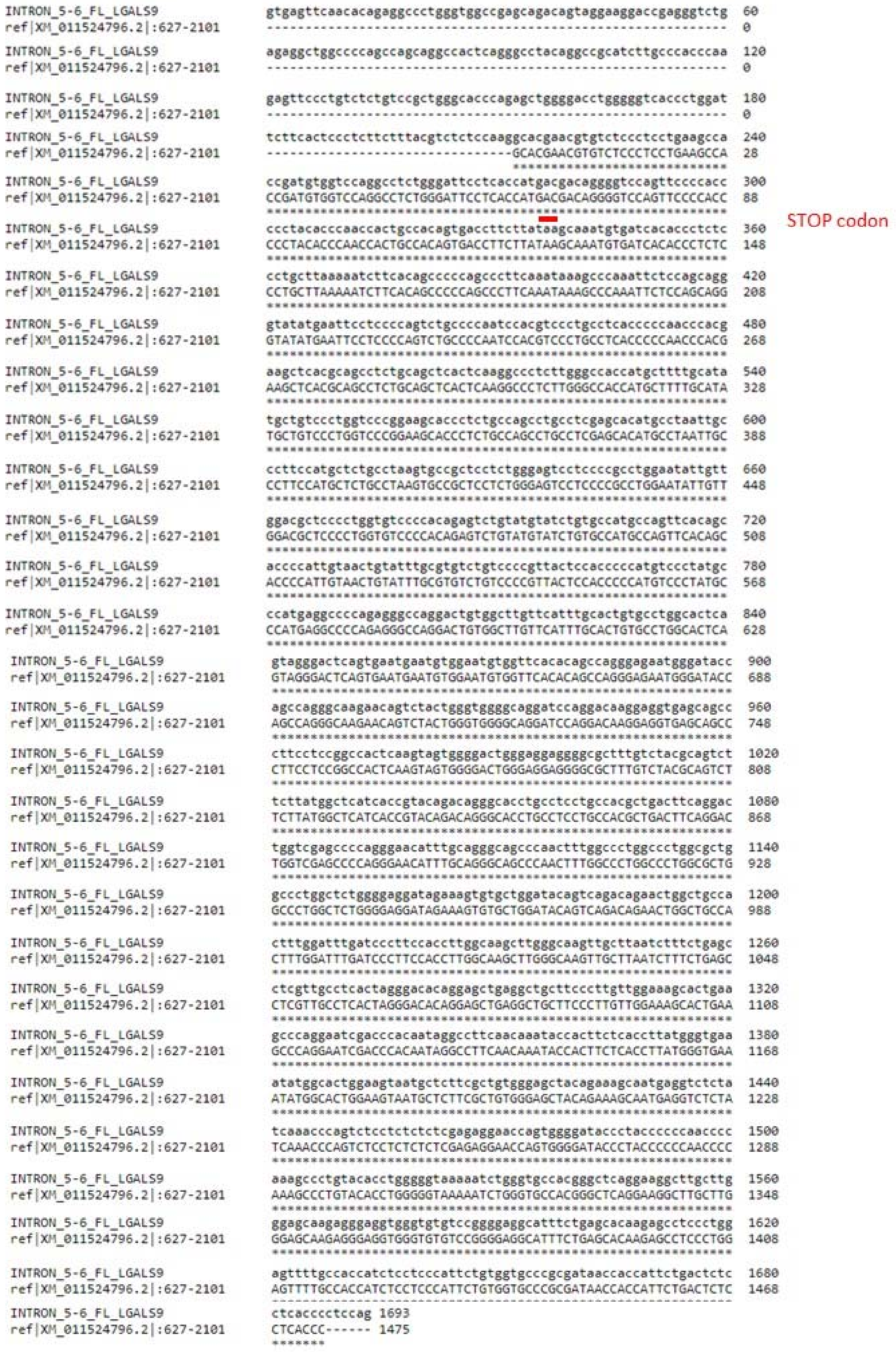

Figure S4: Full alignment (provided by Clustal Omega) of Intron 5-6 from FL *LGALS9* (from database Ensembl) and exon 6´ from X5 variant (from NCBI- GENE database). Intron 5 – 6 of FL is 1693 NTs long, while exon 6´ 1475 NTs. Exon 6´ is almost completely identical with intron 5 – 6, exceptions are only first 212 NTs and last 6 NTS, that are probably spliced out in X5. Higlighted TAA STOP codon which leads to the truncation of X5.

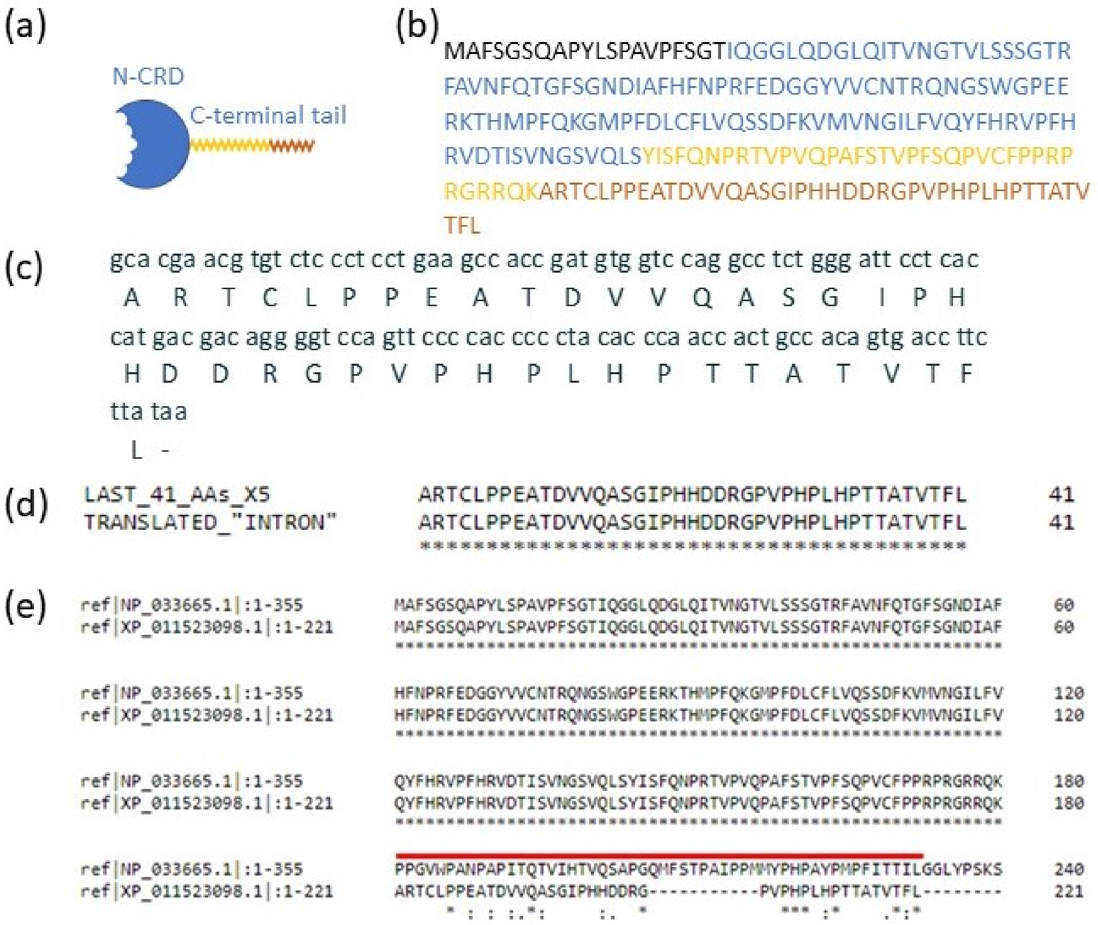

Figure S5: Further characterization of splice variant X5 of GAL-9. (a) predicted protein structure of X5, which probably consists only N-CRD and C-termina peptide tail. (b) ammino acid (AA) sequence of X5 (obtained from NCBI Protein database) with highlighed AA corresponding to each sub-structure of protein. (c) coding sequence of exon 6′ highlighted by red line translated to AAs sequence (Expasy – Translate Tool) (e); d) AAs sequence of "translated" intron 5-6 from FL variant is identical with last 41 AAs of protein sequence of X5 (NCBI- PROTEIN database; analysed by Clustal Omega); e) First 180 AAs of X5 (XP_001523098) are identical with AAs sequence of FL (NP_033665.1).
